# Supplementary material for: Locked-in syndrome in Sweden, an explorative study of persons who underwent rehabilitation: a cohort study
Source: BMJ Open. 2019 Apr 20;9(4):e023185. doi: 10.1136/bmjopen-2018-023185 (PMC6500099; doi:10.1136/bmjopen-2018-023185)
Supplement: Author's manuscript [file bmjopen-2018-023185.draft_revisions.pdf]

BMJ Open is committed to open peer review. As part of this commitment we make the peer review history of every article we publish publicly available.

When an article is published we post the peer reviewers' comments and the authors' responses online. We also post the versions of the paper that were used during peer review. These are the versions that the peer review comments apply to.

The versions of the paper that follow are the versions that were submitted during the peer review process. They are not the versions of record or the final published versions. They should not be cited or distributed as the published version of this manuscript.

BMJ Open is an open access journal and the full, final, typeset and author-corrected version of record of the manuscript is available on our site with no access controls, subscription charges or pay-per-view fees (http://bmjopen.bmj.com).

If you have any questions on BMJ Open's open peer review process please email info.bmjopen@bmj.com

BMJ Open

Locked-in syndrome in Sweden an explorative study of persons who underwent rehabilitation; a cohort study

Journal:	*BMJ Open*
Manuscript ID	bmjopen-2018-023185
Article Type:	Research
Date Submitted by the Author:	27-Mar-2018
Complete List of Authors:	Svernling, Kajsa; Univ of Gothenburg, Inst of Neuroscience and Physiology<br>Törnbom, Marie; Univ of Gothenburg, Inst of Neuroscience and Physiology<br>Nordin, Åsa; Univ of Gothenburg, Inst of Neuroscience and Physiology<br>Sunnerhagen, Katharina; Univ of Gothenburg, Inst of Neuroscience and Physiology
Keywords:	Locked-in syndrome, Living situation, Quality of Life

SCHOLARONE™
Manuscripts

For peer review only

Locked-in syndrome in Sweden an explorative study of persons who underwent rehabilitation; a cohort study

Kajsa Svernling[1], MD, Marie Törnbom[1] , PhD medical social worker, Åsa Nordin[1] RPT, M Sc, Katharina S Sunnerhagen[1*,] MD, PhD

[1]Institute of neuroscience and physiology, department of clinical neuroscience, Sahlgrenska Academy, University of Gothenburg, Gothenburg, Sweden

*Corresponding author:

Katharina S Sunnerhagen

Email address: ks.sunnerhagen@neuro.gu.se

Telephone: +46 709520023

Postal address:

Rehabilitation medicine

Per Dubbsgatan 14, 3rd floor

Sahlgrenska University Hospital

S-413 45 Gothenburg

Sweden

This is an original article written by Kajsa Svernling, Åsa Nordin, Marie Törnbom, and Katharina Stibrant Sunnerhagen. The manuscript is new and is not under consideration by any other journal. Each of the authors has contributed substantially to the manuscript. None of the authors has any financial or non-finacial competing interests. All have read and approved the final version.

Word count Abstract: 263

Word count Main manuscript: 3.935

Number of tables: 7

Number of figures: 1

For peer review only

Abstract

Objective: Locked-in syndrome (LiS) is a rare condition, characterized by a complete paresis except for vertical eye movements and blinking with cognitive functions intact, commonly caused by ischemia in the ventral pons. Previous studies have indicated that persons with LiS can live on for many years and have a good quality of life (QoL). To our knowledge LiS has never been studied in Sweden. The aim was to explore LiS in Sweden; describing population characteristics, living situation, mortality/cause of death and health-related quality of life/impact on participation.

Design: Explorative, nation-wide study in two parts. Persons registered in the national quality register WebRehab during 2007-2014 were eligible.

Participants: Ten persons were identified in part one, four participated in part two. During part one data were collected from WebRehab, medical charts and registers, while questionnaires and interviews were used during part two.

Results: Seven out of ten were men, median age at onset was 49 years and the cause of LiS was in all cases stroke, 70% ischemic. Three were deceased with a median time of survival of 1.9 years. Seven were still alive, with a median time elapsed since onset of 5.8 years. Three participants experienced good quality of life. Information, respect from professionals, and more specialized technical devices were three areas containing unfulfilled needs.

Conclusion: This was the first study conducted in Sweden and the characteristics of this population were like those studied abroad. With proper care, appropriate technical aids and a supportive environment, it is possible for persons with LiS to have a good quality of life but there is still much room for improvements.

Key words: Locked-in syndrome; Living situation; Quality of Life

For peer review only

Strengths and limitations of this study (bullet points)

- The focus of this study was to explore and describe LiS, which has to our knowledge never been studied in Sweden. The study population constituted the whole population of patients with LiS between 2007-2014, no selection has been done from our side.
- We managed to interview four participants of the study population with help from others, and this contributed substantially to the contents of the study.
- This study is limited by its small number of patients and the results can only be indicative and cannot be generalized. Worth noting is that most studies are fewer than ten or even single cases and as such this study does not stand out as small.
- This study was performed in the Swedish context and the results to other cultural contexts needs consideration.

For peer review only

Introduction

LiS is usually caused by a brain stem lesion, most commonly a ventral pontine lesion that interrupts the descending pyramidal tracts (1,3). The lesion is often a result of an ischemic stroke due to thrombosis in the basilar artery, but can also be caused by haemorrhages, trauma, tumors or ischemia due to hypotension (4-6). In rare cases, the cause is metabolic or infectious (7).

The view on prognosis of LiS has shifted a lot through the years. When LiS first became a subject of study, the opinion was that acute mortality was high (2), with nearly no long-term survivors (10). Since then opinion has shifted, numbers on mortality still vary between studies but the overall view on survival is more positive. If the patient medically stabilizes and survives the first year, 5-year survival may be up to 81-86 % (5.11), and some patients survive for decades (7).

Patients with chronic LiS often continue to have highly impaired motor function, even if some improvement is possible (2,11,12). Among other things, the impairments lead to them becoming dependent in the Activities of Daily Living – ADL (self-care etc). Tetraplegia, along with impairments in breathing patterns, also mean respiratory complications are common (2). Most patients living with LiS learn to communicate in some way (7,11,13).

Studies on quality of life – QoL have shown that measured with scales that include motor impairment, LiS patients show lower QoL than healthy controls (14). But measured using scales that do not include motor impairment, their QoL is not significantly altered (14). Mild and moderate depression is more common in LiS patients than healthy controls (14). It is common for patients to be more emotionally sensitive and experience involuntary crying or laughter after the onset of LiS, compared to before (7), a known problem after injuries to the brainstem (15).

For peer review only

Early medical stabilization and early rehabilitation improves prognosis (4,11) and a correct and early diagnosis is essential to minimize suffering and enable proper care (6). When caused by an ischemic stroke, early stroke treatment such as anticoagulation and treatment with tissue plasminogen activator – t-PA could enhance the possibilities of a greater recovery 16,17).

Participation is an aspect of disability which is dependent on both personal and environmental factors, as shown in the ICF-International Classification of Functioning, Disability and Health (18), which is why it becomes an important issue to discuss. Neither autonomy nor participation is static; they are both values that can differ, throughout life (19) which means it can be reduced in one area but still be high in others. This becomes relevant in the topic of LiS, when motor function is low but cognitive function is high.

Severely disabled persons report good QoL despite their serious conditions, which is referred to as the disability paradox (22). Despite researchers belief that the disability paradox was real, it was only when Ubel et al. investigated potential sources of error and explanations of this good QoL, they were able to conclude that it did indeed exist (23This emphasizes the importance of caution for physicians and significant others when forced to make important decisions for their patients or next of kin, and the importance of not easily assuming a low life satisfaction.

The incidence of LiS in Sweden is unknown. Prior to 2015, there was no ICD-code for LiS in ICD-10-SE, which means The National Board of Health and Welfare has no statistics on the syndrome. LiS is not reported in the national quality register for stroke care, Riksstroke (24). One registry in Sweden, WebRehab, offers the possibility to report level of consciousness and thereby report LiS (25). There is a European Network for LiS patients but Sweden is not represented (26).

For peer review only

To our knowledge, LiS has never been researched in Sweden. Previous studies have suggested that rehabilitation in these patients could improve if care was centralized and given by a skilled, professional team (3) and that early, intensive rehabilitation improves prognosis (11). Therefore, there is a need for further research exploring LiS in Sweden, assessing possibilities and needs of that patient population. The aim of this study was to explore and describe LiS in Sweden with the purpose of gaining a better understanding of the life situation for this group of patients.

Material and Methods

This study was performed with material from the registers WebRehab, medical charts, the Swedish Tax Agency's population register, and The National Board of Health and Welfare's registry on Cause of Death (part one) and from questionnaires and interviews (part two).

WebRehab is a National Quality Registry in Rehabilitation Medicine. Twenty-three rehabilitation units in Sweden contribute to the database, representing all 21 counties of Sweden (28). The rehabilitation units report data from the rehabilitation period, including admission and discharge, and from a 1-year follow-up (25). WebRehab is certified at level 2 which is the second highest certification level (29).

Study population

Eligible for this study were persons registered in WebRehab between 2007 - 2014 for whom the level of consciousness according to Giacino (8) was reported. All were diagnosed with locked-in syndrome. Twelve persons were identified from eight different hospitals. Two persons were excluded for not meeting the inclusion criteria. For details see fig 1.

For peer review only

Procedure

This study was approved by the Regional Ethical Review Board in Gothenburg. Application number was 052-15 and was accepted 20150415.

In part one, data from medical charts were gathered for validation and quality control of a quality register. No informed consent from the study participants is required as per Swedish law. In part two, informed, written consent was obtained from all participants or their representative.

During part one data were collected from registers. The identity of the included study participants was obtained from WebRehab´s database. The Functional Independence Measure – FIM – measures level of disability and independence in two scales, one motor scale and one cognitive scale (27) and was analyzed for description of ADL-functions and dependency.

For validation of the data in WebRehab, medical charts were analyzed. To obtain charts, care units were contacted, first by letters, then reminders were sent by email and attempts were made to reach persons in charge by phone.

Data from the Swedish Tax Agency’s population register were collected to investigate how many of the study persons were still alive and to obtain their addresses and contact information.

Data on cause of death and date of death were obtained from The National Board of Health and Welfare’s registry on Cause of Death. Both the application and the communication following the application were written and handled by the first author.

For peer review only

During part two questionnaires and interviews were performed. An information letter along with the questionnaires was sent to all study persons still alive. If no response, a reminder was sent by letter and thereafter attempts to reach the study persons or proxy was by phone. If the study persons wanted to participate, they were asked to send in the questionnaires or contact the author.

Four study persons who agreed to participate were visited for a personal, structured interview. The interviews were conducted in the participants' homes and were recorded and transcribed. All interviews were performed by the first author. During the interviews, some answers were given by a proxy or a personal assistant and the rest were given by the participant and translated by next of kin. After the interview, the participant confirmed that the information given was correct, both the information given in the interview and in the questionnaires. One study participant was not able to perform a personal interview but participated through a telephone interview with her representative

Questionnaires

The different questionnaires used were SIS-v3.0, RAND-36, IPA-E and EQ-5D briefly described below.

SIS-v3.0

To assess health status a Swedish version of the Stroke Impact Scale (SIS-v3.0) (33) was used. The SIS is validated and reliable for use on stroke patients (30).

RAND-36

RAND-36 is a survey instrument that assesses health-related Quality of Life. The Swedish version of RAND-36 is a modern translation of The Short form Health Survey – SF-36, but it

For peer review only - http://bmjopen.bmj.com/site/about/guidelines.xhtml

is similar enough to allow comparisons. It is reliable and valid for measures on HRQoL in stroke patients (31).

IPA-E

IPA-E was used to assess and measure impact on participation and autonomy. The original version of IPA has good validity and reliability (32) IPA-E includes 5 domains, autonomy indoors, family role, autonomy outdoors, social life and relationships, and work and education (32).

EQ-5D

To assess Health Related Quality of Life (HRQoL), a Swedish version of the questionnaire EQ-5D was used. EQ-5D is a standardized, validated health questionnaire developed by the EuroQol Group Association (33). It is intended to be used for self-completion in postal surveys, interviews and clinical practice (33). The EQ-5D is a valid and reliable measure of HRQoL after stroke (34). EQ-5D assesses health in 5 dimensions, mobility, self-care, usual activities, pain/discomfort, and anxiety/depression.

Statistical methods

For statistical analyses IBM® *SPSS Statistics 21* was used. Mainly descriptive statistics with mean and median values were used.

.

For peer review only

Results

Population characteristics

The characteristics of the study population are summarized in table 1.

Table 1 Characteristics of the study population

Study persons	n=10
Alive at 1.5.2015	*7*
Deceased	*3*
Cause of LiS	
Ischemic stroke	*7*
Hemorrhagic stroke	*3*
Classification of LiS at onset	
Classic	*6*
Incomplete	*4*
Previous CVD/VRF	6
Length of Stay Median (Range)	151 days (63-289)
Age at onset Median (Range)	49 y (22-67)
Survival* Median (Range)	1.9 y (1.5-2.3)
Time since onset** Median (Range)	5.9 y (2.3-8.1)

** Deceased study persons included*

*** Alive study persons included*

LiS - Locked-in syndrome, CVD - Cardiovascular disease, VRF - Vascular Risk Factors

For peer review only

Out of ten study persons, there were three women and seven men, age at onset varied from 22 years to 67 years with a median of 49 years. The cause was in all cases a stroke, three were hemorrhagic and seven ischemic. In six of the cases of ischemic stroke, the underlying cause was a basilar thrombosis and the seventh case, a vertebral artery dissection. Six persons had had cardiovascular disease or documented vascular risk factors. The most common vascular

For peer review only - http://bmjopen.bmj.com/site/about/guidelines.xhtml

risk factor was hypertension. Nine of the study persons experienced respiratory complications during hospitalization.

No change in ADL-dependency in motor scale domains was seen in any of the study persons when measured with FIM at admission and discharge; all were totally dependent in all domains. However, FIM showed that five persons improved, four were stable and none deteriorated during rehabilitation; data from one person could not be obtained.

Seven persons from the total population (n=10) were still alive at start of study and three persons were deceased. One person died during rehabilitation and the remaining two after the initial rehabilitation period. Time from onset to date of death varied from 1.5 to 2.3 years with a median of 1.9 years. The cause of death was different for each case: pulmonary embolism, acute myocardial infarction, and acute vascular disorders of the intestine. In none of the cases was the cause of death reported as being the result of respiratory complication due to LiS.

For details of all study persons and of participants, see tables 2 and 3

For peer review only

Table 2. All study persons

	Study persons									
	2	3	4	5	6	7	8	9	10	11
Sex	Female	Female	Male	Female	Male	Male	Male	Male	Male	Male
Age at onset [1]	46 yrs	67 yrs	22 yrs	59 yrs	29 yrs	49 yrs	47 yrs	47 yrs	56 yrs	65 yrs
Etiology	Vascular [illegible]	Vascular [illegible]	Vascular (I)	Vascular [illegible]	Vascular (I)	Vascular (H)	Vascular (I)	Vascular (H)	Vascular (I)	Vascular [illegible]
Classification	Classic	Classic	Incomplete	Classic	Classic	Incomplete	Incomplete	Incomplete	Classic	Classic
Discharged to	Nursing home	Short-term care	Short-term care	Nursing home	Independent living with personal assistance	Independent living with personal assistance		Independent living with personal assistance	Short-term care	Nursing home
Survival	[2]	1.9 y	[2]	2.3 y	[2]	[2]	1.6 y	[2]	[2]	[2]
Cause of death (ICD-10-SE)		Pulmonary embolism (I26.9)		Acute vascular disorders of			Acute myocardial infarction (I21.9)			
Present form of residency	Nursing home		Apartment with community-based support		Independent living with personal assistance	Independent living with personal assistance		Independent living with personal assistance	Independent living with personal assistance	Nursing home

[1] Full years, [2] Still alive

Abbreviations: H - Hemorrhagic, I - Ischemic

For peer review only - http://bmjopen.bmj.com/site/about/guidelines.xhtml

Table 3. Assistance to and communication type of participants

	Participant			
	2	4	6	10
Living arrangement	Lives in a nursing home/home with society-based support. Joint common areas.	Lives in an apartment with society-based support. Lives alone.	Lives in a house. Lives with family.	Lives in a house. Lives with family.
Personal assistance	No personal assistants. Gets assistance from staff.	External personal assistants + extra assistance from staff when needed nighttime	External personal assistance + partner as paid assistant	External personal assistance + partner as paid assistant
Amount	Assistance from staff 168h/week	Assistance - 168 h/week<br>Dual staffing - 10.5 h/week<br>Total – 178.5 h/week	Single staffing - 168 h/week.	Assistance - 168 h/week<br>Dual staffing - 80.5 h/week<br>Total – 248.5 h/week
Communication	Alphabet board, blinking	Alphabet board (blinking)	Oral, alphabet board (pointing)	Blinking, eye-tracking device
Nutrition	Percutaneous endoscopic gastrostomy (PEG)	Oral, pureed/semi-solid	Oral, solid diet	PEG, occasional treat by mouth
Locomotion	Electric wheelchair	Electric wheelchair	Electric/manual wheelchair. Can walk a few steps with support	Electric wheelchair

For peer review only

For peer review only - http://bmjopen.bmj.com/site/about/guidelines.xhtml

HRQoL and impact on participation

Results from the questionnaires are presented separately for each questionnaire. No mean scores were calculated due to the low number of participants. To put the participants' scores in perspective, values for reference populations are included in the tables.

The main finding from the questionnaires is that, although values vary between participants, higher scores were seen in cognitive and mental domains (e.g. SIS-v3.0 Memory and Emotion and RAND-36 Mental Health) than in physical domains (e.g. SIS-v3.0 Strength, RAND-36 Physical Functioning and EQ-5D Mobility).

Individual scores on the SIS-v3.0 are presented in table 4, on RAND-36 in table 5, on IPA-E in table 6 and on EQ-5D in table 7.

Table 4 Stroke Impact Scale Results

	Individual score				Reference*
**Domain**	**2**	**4**	**6**	**10**	Mean *(SD)*
Strength	25	37.5	25	0	71 *(26.9)*
Hand function	0	0	30	0	81.1 *(22.2)*
Mobility	0	0	19.4	5.6	77.6 *(17.4)*
ADL	0	12.5	15	20	85 *(21)*
Emotion	44.4	94.4	88.9	86.1	75 *(28.5)*
Memory	100	42.9	100	71.4	75.6 *(29.2)*
Communication	75	85.7	53.6	14.3	68.9 *(34.5)*
Social participation	12.5	31.25	78.1	25	70.3 *(27.5)*
Stroke recovery	20	50	100	10	68.4 *(25.5)*

* Swedish stroke population, assessed 12 months after stroke (38).

For peer review only - http://bmjopen.bmj.com/site/about/guidelines.xhtml

Table 5 RAND-36 Results

Domain	Individual score 2	4	6	10	Reference (LiS)* Mean *(SD)*	Reference (Stroke)** Mean *(95% CI)*
Physical functioning	0	0	5	0	0 *(0)*	51.2 *(44.3-58.1)*
Role limitations due to physical health	0	0	100	0	59.4 *(32.6)*	14.7 *(3.3-26.1)*
Role limitations due to emotional problems	33.3	100	100	100	75.0 *(34.5)*	18.0 *(10.2-25.8)*
Vitality/Energy-fatigue	45	55	75	70	64.4 *(24.6)*	42.9 *(37.8-48.0)*
Mental health/Emotional well-being	60	76	92	76	68 *(19.6)*	62.7 *(58.2-67.2)*
Social functioning	12.5	75	50	87.5	56.3 *(34.1)*	55.2 *(49.2-61.4)*
Bodily pain	80	90	100	67.5	82 *(26.8)*	65.0 *(57.9-72.1)*
General health	40	30	100	60	63.5 *(33.0)*	58.2 *(52.5-63.9)*

* Belgian LiS population, assessed more than 12 months after onset (12).
** Swedish stroke population, assessed 2 years after day hospital rehabilitation for stroke (39).

Table 6 Impact on Participation and Autonomy Questionnaire Results

Domain	Individual score 2	4	6	10	Reference * Mean *(SD)*
Autonomy indoors	4	3	0	2	0.96 *(0.6)*
Family role	4	4	4	4	1.96 *(1.1)*
Autonomy outdoors	4	3	2	3	2.35 *(0.9)*
Social life and relations	3	2	3	3	1.48 *(0.7)*
Work and education	-[1]	-[1]	1	-[1]	-

[1]Cannot be calculated, participant is not currently employed.
*Iranian stroke population, assessed 5-36 months after their stroke (40).

For peer review only - http://bmjopen.bmj.com/site/about/guidelines.xhtml

Table 7 EuroQol-5 dimensions Results

	7ndividual score				Reference*
**Domain**	**2**	**4**	**6**	**10**	Mean *(95% CI)*
Mobility	3	3	3	3	-
Self-care	3	3	3	3	-
Usual activities	3	3	1	3	-
Pain/discomfort	3	2	1	2	-
Anxiety/depression	2	2	1	2	-
Index value	-0.429	-0.166	0.122	-0.166	0.44 *(0.28-0.42)*
VAS	0.02	0.5	1	0.4	0.63 *(58.8-66.6)*

*Swedish stroke population, assessed 2 years after day hospital rehabilitation for stroke (40).

Interviews

Information

In all but one interview, participants described how there was a lack of information about LiS and it was also difficult to get information about technical devices, or the follow-up procedure. They mentioned that they were sometimes forced to find technical aids and organize follow-up on their own. One participant, and a proxy, intended to write a book for persons with LiS about how to get information and support. This information and support was also found by this participant by becoming a member of a neurological association.

When participants were in intensive care, they felt they got better information than after discharge, although medical terms were sometimes difficult to understand.

Technical devices, technical adjustments and shortcomings

All used one or more wheel-chair/s, alphabet boards and used or wanted to use an eye-tracking computer device. This kind of computer is expensive and was not available for everyone.

For peer review only - http://bmjopen.bmj.com/site/about/guidelines.xhtml

An alarm button which can be placed by the temple, adjusted hospital beds and lifts, tilting table, ceiling hoist, a walking frame with extra support and a sit-to stand and transfer device were also mentioned as technical devices that participants had access to. Two used bicycles for passive cycling. One person wanted a Functional Electric Stimulation/FES-assisted training device but was refused grants from the municipality to fund it. This person was also part of a customer test group for a device which combines eye-tracking technology with an electric wheelchair to enable control of the wheelchair with eye movements. He was promised money to purchase a wheelchair because of his help in developing the new technology.

Adjustments of homes were made such as installing ramps, widening doors, thresholds and elevated toilet seats.

Progress after rehabilitation and care

Several years after stroke, participants' motor functions were still improving. They could communicate with the help of an alphabet board and used an eye-tracking computer device, or communicated mainly by blinking. One person had some oral communication including most vowels, some consonants and a few short words, today he can control his jaw and tongue muscles and he has some movement in his fingers. Another could walk a few steps with support, lift his arms and move his head; he also had some function in his left hand, but was still completely dependent in ADL. Two persons could eat solid food and had no additional nutritional support. One person had an alarm button with a pre-recorded sentence which he could press by turning his head to attract attention. During the last year, he had learnt to shake his head and is currently practicing nodding. All needed a lot of daily support and had personal assistants around the clock and sometimes two assistants at the same time. Additional help from a nursing home was also reported.

For peer review only - http://bmjopen.bmj.com/site/about/guidelines.xhtml

Likes and dislikes about care and authorities

They appreciated having phenomenal physiotherapists. Every second week both an occupational therapist and a physiotherapist visited one participant, she still wanted a lot more additional training on top of these visits. Some said contacts with authorities mostly worked well, although it could be time-consuming, but one argued that daily contacts with caregivers worked less well. One participant felt unhappy because she was in a nursing home and could not live on her own terms but was granted a flat of her own in due time.

Some participants/ proxies were offered support by a counsellor and were pleased with this. One partner talked about the difficult mourning process after LiS. Nothing turned out the way she had wanted because of her husband´s LiS. She was helped to think and reflect in a new way, like what could be planned and done despite LiS. One partner reflected that during intense rehabilitation periods one gets a lot of support from all specialists but then it ends, for example appointments with a counsellor. One person wanted to meet a specialist doctor but felt he was not heard, having so many questions not answered by the local doctor.

Attitudes from professionals

All experienced not being treated as adults, people talked to them slowly and loudly as if they were not able to understand anything which was frustrating. This could be aggravating if a person treating you did not know you well enough and/or did not have enough knowledge about the syndrome. A proxy said that just being able to nod your head led to a participant being treated with more respect.

For peer review only - http://bmjopen.bmj.com/site/about/guidelines.xhtml

Family and friends

One participant had separated from her husband and had a complicated life situation. The others had mostly good family relationships, sometimes wives worked as personal assistants. It was articulated that a relationship with a daughter had worsened after LiS and that this was the worst thing that had happened. Friends might have disappeared after LiS but the remaining ones were appreciated.

For peer review only

Quality of life

All but one evaluated their current life situation. They mentioned feeling joy at being alive and having the ability to encourage others. Improving quality of life was, for example, being granted more hours with personal assistants or getting in contact with a person with the same syndrome.

Discussion

Findings

We identified ten persons who had been diagnosed with LiS between 2007 and 2014 and investigated factors at onset, during the rehabilitation period, and after discharge. Seven of these persons were still alive at the start of the study and four of these participated. The common cause was stroke and most of these were ischemic. According to medical charts, some of the study persons improved in their motor function and all are still improving, although not in independency measured with the FIM motor scale. No major cognitive deficits were reported. Mortality in this population was 30 % and mean survival time for the deceased was 1.9 years. For the study persons still alive, mean time since onset of LiS was 5.9 years. From the interviews we identified areas with unfulfilled needs; information, respect, and specialized technical aids. The data from the questionnaires were mostly in line with

For peer review only - http://bmjopen.bmj.com/site/about/guidelines.xhtml

information from the interviews, those who expressed high QoL and high impact on participation and autonomy in the interview, also scored high in domains not affected by motor impairments in the questionnaires.

Strengths and limitations

Since this was a nation-wide study and no selection was done, the number of patients might be considered few compared to previous studies abroad with study samples of around 20-30 persons (5, 7, 14). One hypothesis could be that incidence numbers on LiS differ for some reason in Sweden. Another could be that incidence numbers are similar but all persons with LiS in Sweden have not been identified. This study population was identified through a register for rehabilitation medicine and did not include patients who died in intensive care units. Up until the end of 2014, WebRehab was, to our knowledge, the only register in Sweden that had statistics on LiS but as of 1st of January 2015, there is an ICD-code in ICD-10-SE (35). What this will mean for the care of LiS-patients is hard to predict but reporting LiS will be possible for every care unit. Another possible explanation for the small number of persons identified is that patients with LiS were not diagnosed and registered.

The questionnaires used are similar to each other in many ways, but all of them include unique aspects when compared to each other. EQ-5D is a quite rough instrument and physical functions have a large impact on the result. The value of using it in this particular study, where physical functions are very low, might therefore be worth discussing. In the EQ-5D, an index score under 0 is described as indicating a condition worse than death which is a problematic statement, e.g. three of our participants had an index score under 0, none of them described their situation as worse than death.

When presenting the results of the questionnaires, values from reference populations are included in the tables, and our reasoning was to put our participants' scores in perspective. But due to the low numbers of participants, no further comparisons can be made. The

For peer review only - http://bmjopen.bmj.com/site/about/guidelines.xhtml

reference groups are all stroke populations, because all our participants had suffered a stroke and are included in this patient group.

The method chosen for this study was a quantitative, descriptive method containing analysis of register data, medical charts and structured interviews based on questionnaires. The reason we chose this approach instead of a qualitative method with unstructured interviews was the participants' limited ability to effectively communicate, which we believe would mean unstructured interviews would not give more information than structured interviews.

By combining questionnaires with personal interviews, the participants had a chance to elaborate on their answers and opinions while still maintaining a standardized form of assessment with the questionnaires.

Since a proxy or a personal assistant was present and involved in the interviews, there is a risk of misinterpretations in translations or that things did not get relayed in the way the participant intended. All participants were therefore asked if the information given was correct at the end of the interviews.

Comparison with existing literature and guidelines

It seems consistent with previous knowledge that mortality is high during the first years after onset but when stabilized, persons with LiS may live for decades (7). Since onset of LiS for our study population only goes back to 2007, long term survival cannot be commented upon here.

The cause of death of the study persons was never a result of respiratory complications or problems with breathing, despite the high prevalence of respiratory complications during hospitalization. This might be explained by proper care which has prevented or successfully treated any respiratory problems, but it might also be explained by simple luck.

The importance of maintaining vital functions and that fatality in LiS have declined with improvements in quality of medicine has been discussed in previous studies (6). So has the

For peer review only - http://bmjopen.bmj.com/site/about/guidelines.xhtml

fact that LiS can be mistaken for other Disorders of Consciousness - DOC when not assessed properly or thoroughly enough (9). A missed diagnosis could therefore be due to, e.g., vital functions not being maintained or that the condition was misdiagnosed as another DOC.

In line with previous findings (7, 11, 12), most of the study persons were middle-aged with a median age of 49 years at onset. In line with our study, patients with chronic LiS have slim chances of major improvements in motor function (11).

According to FIM, five persons improved in independency in cognitive domains. Expression and problem solving are the two areas with the least improvements which might be explained by the poor communication skills of this patient group.

In many aspects the results on the questionnaires varied between the participants but most of them scored high on domains measuring emotional and cognitive functions and low on physical domains and functions. This is in line with previous knowledge that QoL in persons with LiS can be high scoring in mental domains, while low scoring in physical domains (14).

Clinical and policy implications

Problems like nursing staff using complicated medical terminology or not getting enough information about technical and rehabilitation aids were brought up. As this patient group has limited communication skills, it seems important to fulfil all their needs for technical equipment to improve their QoL. All participants had experienced not being treated with respect and felt that people talked over their heads. These problems have been discussed in context of disability and aphasia, it affects social participation and with that, QoL in a negative way (36, 37). Raising awareness about LiS, both among caregivers and the public is necessary, since it might lead to less insecurity and incertitude when meeting a person with LiS and hopefully, therefore result in people treating a person with LiS with more respect.

For peer review only - http://bmjopen.bmj.com/site/about/guidelines.xhtml

Future research

From 2015 there will be an ICD-code in ICD-10-SE, which will improve the possibility of longitudinal research of this group of patients in Sweden. This seems to be an urgent field of research because, to our knowledge, there are no articles yet from Sweden about LiS. Also, improving the development and research of new technical devices for this group of patients might help them to communicate more efficiently and to find methods of improving motor functions.

Author contribution

KS, ÅN and KSS contributed to the design of the study. KS met the participants and gave them questionnaires, conducted and summarized the structured interviews. MT categorized the interviews inductively and wrote the manuscript together with KS and KSS.

Data sharing statement

Due to ethical restrictions data are available upon request. Interested researchers may submit requests for data to the authors (contact Professor Katharina S. Sunnerhagen, email: ks.sunnerhagen@neuro.gu.se). According to Swedish regulation: http://www.epn.se/en/start/regulations/, the permission to use data is only for what has been applied for and then approved by the Ethical board. To not follow the regulations is seen as scientific misconduct.

Competing interests

All authors declare that there is no conflict of interest.

Acknowledgements

We would like to acknowledge the help from WebRehab and the clinics that provided information on the persons. At last but not least, we would like to to thank the participants for

For peer review only - http://bmjopen.bmj.com/site/about/guidelines.xhtml

For peer review only

answering questionnaires. A sincere thanks to them and their families for allowing KS to visit them and for generously telling about their situation and experiences. We would like to thank Dr Kate Bramley-Moore for her English language assistance and proofreading.

Funding

The study is in part funded by The Swedish Research Council (2017-00946) and a grant from the Swedish Heart and Lung Foundation (20160526). The supporting bodies had no influence on the content of the study.

Patient and Public Involvement

In the steering committee for the national quality register WebRehab, there are two representatives from patient organizations. There were no patient representatives involved in the recruitment to or conduction of the study. A short summary of the results have been distributed to the participants in the interviews.

For peer review only

For peer review only - http://bmjopen.bmj.com/site/about/guidelines.xhtml

References

1. Posner JB, Saper CB, Schiff ND, Plum F. Plum and Posner's Diagnosis of Stupor and Coma. 4 ed. New York: Oxford University Press; 2007.
2. Patterson JR, Grabois M. Locked-in syndrome: a review of 139 cases. Stroke. Jul-Aug 1986;17(4):758-764.
3. Schjolberg A, Sunnerhagen KS. Unlocking the locked in; a need for team approach in rehabilitation of survivors with locked-in syndrome. Acta Neurol Scand. Mar 2012;125(3):192-198.
4. Doble JE, Haig AJ, Anderson C, Katz R. Impairment, activity, participation, life satisfaction, and survival in persons with locked-in syndrome for over a decade: follow-up on a previously reported cohort. J Head Trauma Rehabil. Sep-Oct 2003;18(5):435-444.
5. Katz RT, Haig AJ, Clark BB, DiPaola RJ. Long-term survival, prognosis, and life-care planning for 29 patients with chronic locked-in syndrome. Arch Phys Med Rehabil. May 1992;73(5):403-408.
6. Nikic PM, Jovanovic D, Paspalj D, Georgievski-Brkic B, Savic M. Clinical characteristics and outcome in the acute phase of ischemic locked-in syndrome: case series of twenty patients with ischemic LIS. Eur Neurol. 2013;69(4):207-212.
7. Leon-Carrion J, van Eeckhout P, Dominguez-Morales Mdel R, Perez-Santamaria FJ. The locked-in syndrome: a syndrome looking for a therapy. Brain Inj. Jul 2002;16(7):571-582.
8. Giacino JT, Ashwal S, Childs N, et al. The minimally conscious state: Definition and diagnostic criteria. Neurology. 2002;58(3):349-353.
9. Kotchoubey B, Lotze M. Instrumental methods in the diagnostics of locked-in syndrome. Restor Neurol Neurosci. 2013;31(1):25-40.
10. Pearce JM. The locked in syndrome. Br Med J (Clin Res Ed). Jan 24 1987;294(6566):198-199.
11. Casanova E, Lazzari RE, Lotta S, Mazzucchi A. Locked-in syndrome: improvement in the prognosis after an early intensive multidisciplinary rehabilitation. Arch Phys Med Rehabil. Jun 2003;84(6):862-867.
12. Snoeys L, Vanhoof G, Manders E. Living with locked-in syndrome: an explorative study on health care situation, communication and quality of life. Disabil Rehabil. May 2013;35(9):713-718.
13. Soderholm S, Meinander M, Alaranta H. Augmentative and alternative communication methods in locked-in syndrome. J Rehabil Med. Sep 2001;33(5):235-239.
14. Rousseau MC, Pietra S, Nadji M, Billette De Villemeur T. Evaluation of quality of life in complete locked-in syndrome patients. Journal of Palliative Medicine. 2013;16(11):1455-1458.
15. Parvizi J, Anderson SW, Martin CO, Damasio H, Damasio AR. Pathological laughter and crying: A link to the cerebellum. Brain. 2001;124(9):1708-1719.
16. Barbic D, Levine Z, Tampieri D, Teitelbau J. Locked-in syndrome: a critical and time-dependent diagnosis. CJEM. Sep 2012;14(5):317-320.
17. Janjua N, Wartenberg KE, Meyers PM, Mayer SA. Reversal of locked-in syndrome with anticoagulation, induced hypertension, and intravenous t-PA. Neurocrit Care. 2005;2(3):296-299.
18. World Health Organization. International Classification of Functioning, Disability and Health (ICF). Geneva: World Health Organization; 2001.
19. Cardol M, De Jong BA, Ward CD. On autonomy and participation in rehabilitation. Disability and Rehabilitation. 2002;24(18):970-974.
20. Yeung P, Towers A. An exploratory study examining the relationships between the personal, environmental and activity participation variables and quality of life among young adults with disabilities. Disability and Rehabilitation. 2014;36(1):63-73.

For peer review only - http://bmjopen.bmj.com/site/about/guidelines.xhtml

21. Kersten P, Cardol M, George S, Ward C, Sibley A, White B. Validity of the impact on participation and autonomy questionnaire: a comparison between two countries. Disabil Rehabil. Oct 15 2007;29(19):1502-1509
22. Albrecht GL, Devlieger PJ. The disability paradox: High quality of life against all odds. Social Science and Medicine. 1999;48(8):977-988.
23. Ubel PA, Schwarz N, Loewenstein G, Smith D. Misimagining the unimaginable: The disability paradox and health care decision making. Health Psychology. 2005;24(4 SUPPL.):S57-S6
24. Asplund K, Hulter Asberg K, Appelros P, et al. The Riks-Stroke story: building a sustainable national register for quality assessment of stroke care. Int J Stroke. Apr 2011;6(2):99-108.
25. Sunnerhagen KS, Flansbjer UB, Lannsjo M, Tolli A, Lundgren-Nilsson A. WebRehab: a Swedish database for quality control in rehabilitation. J Rehabil Med. Nov 4 2014;46(10):958-962.
26. Association du Locked in Syndrome ALIS. LISEF LIS European Foundation. [Internet] [cited 2015 May 26th]; Available from: http://www.alis-asso.fr/lisef/.
27. Grimby G, Gudjonsson G, Rodhe M, Sunnerhagen KS, Sundh V, Ostensson ML. The functional independence measure in Sweden: experience for outcome measurement in rehabilitation medicine. Scand J Rehabil Med. May 1996;28(2):51-62.
28. Om WebRehab Sweden. [Internet] Uppsala: WebRehab Sweden; [updated 2014 Apr 3 cited 2015 Feb 2]; Available from: http://www.ucr.uu.se/webrehab/index.php/om-webrehab-sweden
29. Nationella kvalitetsregister, Certifieringsnivåer. [Internet] Stockholm: Sveriges Kommuner och Landsting,; [updated 2014, March 26; cited 2015 Feb 12]; Available from: http://www.kvalitetsregister.se/registerarbete/certifieringsnivaer.
30. Duncan PW, Wallace D, Lai SM, Johnson D, Embretson S, Laster LJ. The stroke impact scale version 2.0. Evaluation of reliability, validity, and sensitivity to change. Stroke. Oct 1999;30(10):2131-2140.
31. Anderson C, Laubscher S, Burns R. Validation of the Short Form 36 (SF-36) health survey questionnaire among stroke patients. Stroke. 1996;27(10):1812-1816.
32. Lund ML, Fisher AG, Lexell J, Bernspang B. Impact on participation and autonomy questionnaire: internal scale validity of the Swedish version for use in people with spinal cord injury. J Rehabil Med. Mar 2007;39(2):156-162.
33. EuroQol Research Foundation. [Internet] Rotterdam: EuroQol Research Foundation; 2015 [cited 2015 Feb 6]; Available from: http://www.euroqol.org/about-eq-5d.html
34. Dorman PJ, Waddell F, Slattery J, Dennis M, Sandercock P. Is the EuroQol a valid measure of health-related quality of life after stroke? Stroke. Oct 1997;28(10):1876-18
35. Socialstyrelsen. Ändringar i och tillägg till Internationell statistisk klassifikation av sjukdomar och relaterade hälsoproblem - systematisk förteckning, svensk version 2011 (ICD-10-SE) gällande från den 1 januari 2015 2014-10-03.
36. Dalemans RJ, de Witte L, Wade D, van den Heuvel W. Social participation through the eyes of people with aphasia. Int J Lang Commun Disord. Sep-Oct 2010;45(5):537-550.
37. Wagner M. "Does he take sugar?". World Health Forum. 1991;12(1):87-89.
38. Guidetti S, Ytterberg C, Ekstam L, Johansson U, Eriksson G. Changes in the impact of stroke between 3 and 12 months post-stroke, assessed with the Stroke Impact Scale. J Rehabil Med. Nov 2014;46(10):963-968.
39. Grenthe Olsson B, Sunnerhagen KS. Functional and cognitive capacity and health-related quality of life 2 years after day hospital rehabilitation for stroke: a prospective study. J Stroke Cerebrovasc Dis. Sep-Oct 2007;16(5):208-215.
40. Fallahpour M, Tham K, Joghataei MT, Jonsson H. Perceived participation and autonomy: aspects of functioning and contextual factors predicting participation after stroke. J Rehabil Med. Apr 2011;43(5):388-397.

For peer review only - http://bmjopen.bmj.com/site/about/guidelines.xhtml

Figure 1 legend

Flow chart of the study. One person was excluded after the validation process due to not meeting the inclusion criteria (locked in syndrome) and one person due to not having a valid personal identity number.

For peer review only

For peer review only - http://bmjopen.bmj.com/site/about/guidelines.xhtml

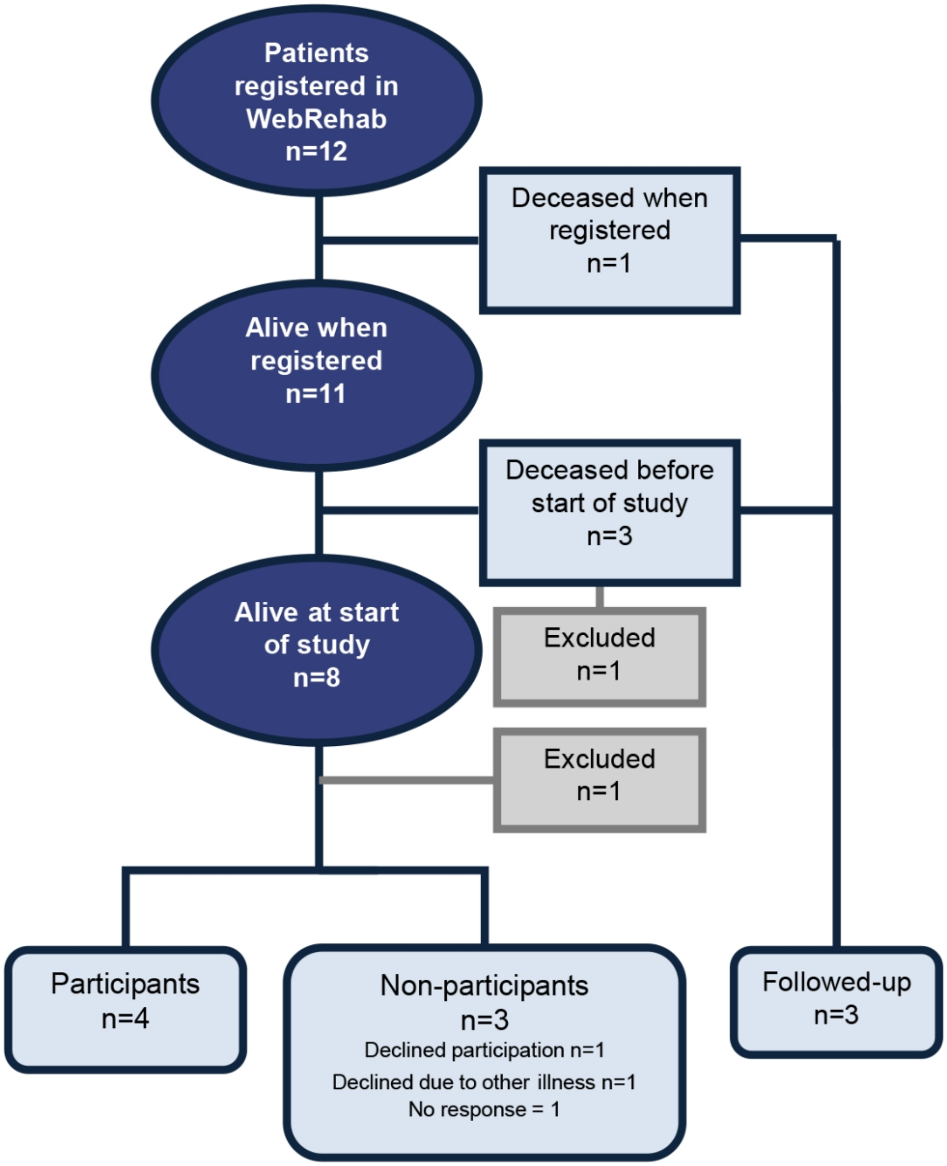

99x119mm (300 x 300 DPI)

STROBE 2007 (v4) Statement—Checklist of items that should be included in reports of *cohort studies*

Section/Topic	Item #	Recommendation	Reported on page #
**Title and abstract**	1	(*a*) Indicate the study's design with a commonly used term in the title or the abstract	1
		(*b*) Provide in the abstract an informative and balanced summary of what was done and what was found	2
**Introduction**			
Background/rationale	2	Explain the scientific background and rationale for the investigation being reported	4,5
Objectives	3	State specific objectives, including any prespecified hypotheses	6
**Methods**			
Study design	4	Present key elements of study design early in the paper	6
Setting	5	Describe the setting, locations, and relevant dates, including periods of recruitment, exposure, follow-up, and data collection	6,7
Participants	6	(*a*) Give the eligibility criteria, and the sources and methods of selection of participants. Describe methods of follow-up	7
		(*b*) For matched studies, give matching criteria and number of exposed and unexposed	NA
Variables	7	Clearly define all outcomes, exposures, predictors, potential confounders, and effect modifiers. Give diagnostic criteria, if applicable6	8.9
Data sources/ measurement	8*	For each variable of interest, give sources of data and details of methods of assessment (measurement). Describe comparability of assessment methods if there is more than one group	*8,9*
Bias	9	Describe any efforts to address potential sources of bias	NA
Study size	10	Explain how the study size was arrived at	8,9
Quantitative variables	11	Explain how quantitative variables were handled in the analyses. If applicable, describe which groupings were chosen and why	9
Statistical methods	12	(*a*) Describe all statistical methods, including those used to control for confounding	9
		(*b*) Describe any methods used to examine subgroups and interactions	NA
		(*c*) Explain how missing data were addressed	NA
		(*d*) If applicable, explain how loss to follow-up was addressed	NA
		(*e*) Describe any sensitivity analyses	NA
**Results**			

For peer review only

Participants	13*	(a) Report numbers of individuals at each stage of study—eg numbers potentially eligible, examined for eligibility, confirmed eligible, included in the study, completing follow-up, and analysed	9
		(b) Give reasons for non-participation at each stage	9
		(c) Consider use of a flow diagram	Fig 1
Descriptive data	14*	(a) Give characteristics of study participants (eg demographic, clinical, social) and information on exposures and potential confounders	10,12
		(b) Indicate number of participants with missing data for each variable of interest	10,12
		(c) Summarise follow-up time (eg, average and total amount)	
Outcome data	15*	Report numbers of outcome events or summary measures over time	12, 14.15,16
Main results	16	(*a*) Give unadjusted estimates and, if applicable, confounder-adjusted estimates and their precision (eg, 95% confidence interval). Make clear which confounders were adjusted for and why they were included	
		(*b*) Report category boundaries when continuous variables were categorized	
		(*c*) If relevant, consider translating estimates of relative risk into absolute risk for a meaningful time period	
Other analyses	17	Report other analyses done—eg analyses of subgroups and interactions, and sensitivity analyses	16,17
**Discussion**			
Key results	18	Summarise key results with reference to study objectives	20
**Limitations**			
Interpretation	20	Give a cautious overall interpretation of results considering objectives, limitations, multiplicity of analyses, results from similar studies, and other relevant evidence	20
Generalisability	21	Discuss the generalisability (external validity) of the study results	20
**Other information**			
Funding	22	Give the source of funding and the role of the funders for the present study and, if applicable, for the original study on which the present article is based	24

*Give information separately for cases and controls in case-control studies and, if applicable, for exposed and unexposed groups in cohort and cross-sectional studies.

Note: An Explanation and Elaboration article discusses each checklist item and gives methodological background and published examples of transparent reporting. The STROBE checklist is best used in conjunction with this article (freely available on the Web sites of PLoS Medicine at http://www.plosmedicine.org/, Annals of Internal Medicine at http://www.annals.org/, and Epidemiology at http://www.epidem.com/). Information on the STROBE Initiative is available at www.strobe-statement.org.

For peer review only

BMJ Open

Locked-in syndrome in Sweden an explorative study of persons who underwent rehabilitation; a cohort study

Journal:	*BMJ Open*
Manuscript ID	bmjopen-2018-023185.R1
Article Type:	Research
Date Submitted by the Author:	29-Nov-2018

Complete List of Authors:	Svernling, Kajsa; Univ of Gothenburg, Inst of Neuroscience and Physiology<br>Törnbom, Marie; Univ of Gothenburg, Inst of Neuroscience and Physiology<br>Nordin, Åsa; Univ of Gothenburg, Inst of Neuroscience and Physiology<br>Sunnerhagen, Katharina; Univ of Gothenburg, Inst of Neuroscience and Physiology

**Primary Subject Heading**:	Neurology
Secondary Subject Heading:	Rehabilitation medicine
Keywords:	Locked-in syndrome, Living situation, Quality of Life

SCHOLARONE™
Manuscripts

Locked-in syndrome in Sweden an explorative study of persons who underwent rehabilitation; a cohort study

Kajsa Svernling[1], MD, Marie Törnbom[1] , PhD medical social worker, Åsa Nordin[1] RPT, M Sc, Katharina S Sunnerhagen[1*], MD, PhD

[1]Institute of neuroscience and physiology, department of clinical neuroscience,

Sahlgrenska Academy, University of Gothenburg, Gothenburg, Sweden

*Corresponding author:

Katharina S Sunnerhagen

Email address: ks.sunnerhagen@neuro.gu.se

Telephone: +46 709520023

Postal address:

Rehabilitation medicine

Per Dubbsgatan 14, 3rd floor

Sahlgrenska University Hospital

S-413 45 Gothenburg

Sweden

This is an original article written by Kajsa Svernling, Åsa Nordin, Marie Törnbom, and Katharina Stibrant Sunnerhagen. The manuscript is new and is not under consideration by any other journal. Each of the authors has contributed substantially to the manuscript. None of the authors has any financial or non-financial competing interests. All have read and approved the final version.

Word count Abstract: 263

Word count Main manuscript: 3.935

Number of tables: 7

Number of figures: 1

For peer review only

Abstract

Objective: Locked-in syndrome (LiS) is a rare condition, characterized by a complete paresis except for vertical eye movements and blinking with cognitive functions intact, commonly caused by ischemia in the ventral pons. Previous studies have indicated that persons with LiS can live on for many years and have a good quality of life (QoL). To our knowledge, LiS has never been studied in Sweden. The aim was to explore LiS in Sweden; describing population characteristics, living situation, mortality/cause of death and health-related quality of life/impact on participation.

Design: Explorative, nation-wide study in two parts. Persons registered in the national quality register WebRehab during 2007-2014 were eligible.

Participants: Ten persons were identified in part one, four participated in part two. During part one data were collected from WebRehab, medical charts and registers, while questionnaires and interviews were used during part two.

Results: Seven out of ten were men, median age at onset was 49 years and the cause of LiS was in all cases stroke, 70% ischemic. Three were deceased with a median time of survival of 1.9 years. Seven were still alive, with a median time elapsed since onset of 5.8 years. Three participants experienced good quality of life. Information, respect from professionals, and more specialized technical devices were three areas containing unfulfilled needs.

Conclusion: This was the first study conducted in Sweden and the characteristics of this population were like those studied abroad. In this study, the persons with LiS that were interviewed expressed the need for proper care, appropriate technical aids and a supportive environment in order to have quality of life. However, there is still much room for improvements.

For peer review only - http://bmjopen.bmj.com/site/about/guidelines.xhtml

Key words: Locked-in syndrome; Living situation; Quality of Life

Strengths and limitations of this study (bullet points)

- The focus of this study was to explore and describe LiS, which has to our knowledge never been studied in Sweden. The study population constituted the whole population of patients with LiS receiving in-patient rehabilitation between 2007-2014.
- We managed to interview four participants of the study population with help from others, and this contributed substantially to the contents of the study.
- This study is limited by its small number of patients and the results can only be indicative and cannot be generalized. Worth noting is that most studies are fewer than ten or even single cases and as such, this study does not stand out as small.
- This study was performed in the Swedish context and the results to other cultural contexts needs consideration.

For peer review only

Introduction

Locked in Syndrome (LiS) is a state in which a patient is both quadriplegic and paralyzed to the lower cranial nerves but conscious and with retained control of vertical eye movement and eyelids. LiS is usually caused by a brain stem lesion, most commonly a ventral pontine lesion that interrupts the descending pyramidal tracts.[1-3] The lesion is often a result of an ischemic stroke due to thrombosis in the basilar artery, but can also be caused by haemorrhages, trauma, tumours or ischemia due to hypotension.[4-6] In rare cases, the cause is metabolic or infectious.[7]

The American Congress of Rehabilitation Medicine[8] recommends neurobehavioral criteria to be used when diagnosing LiS. The criteria are 1: Eye opening well sustained, 2: Basic cognitive abilities preserved (clinical examination), 3: Severe hypophonia or apohonia on clinical examination, 4: Quadriplegia/Quadriparesis on clinical examination and 5: Communication primarily through eye movements or through blinking. The LiS can vary in clinical presentation. *Classic LiS* is defined as a fully paralyzed patient with intact vertical eye movements and movement in the eyelid. *Incomplete LiS* is similar to Classic LiS but with remnants of motor functions beyond those of the classic variant, usually some movement in fingers and/or toes. *Total LiS* is defined as total immobility, the use of electroencephalography – EEG is then necessary to ascertain consciousness. In context of duration, LiS can be chronic or transient, in the latter the patient recovers completely.

For peer review only - http://bmjopen.bmj.com/site/about/guidelines.xhtml

The view on prognosis of LiS has shifted a lot through the years. When LiS first became a subject of study, the opinion was that acute mortality was high,[2] with nearly no long-term survivors.[9] Since then opinion has shifted, numbers on mortality still vary between studies but the overall view on survival is more positive. If the patient medically stabilizes and survives the first year, 5-year survival may be up to 81-86 %,[5 10] and some patients survive for decades.[7]

Patients with chronic LiS often continue to have highly impaired motor function, even if some improvement is possible.[3 10 11] Among other things, the impairments lead to them becoming dependent in the Activities of Daily Living – ADL (self-care etc.). Tetraplegia, along with impairments in breathing patterns, also mean respiratory complications are common.[3] Most patients living with LiS learn to communicate in some way.[7 10 12]

Studies on quality of life – QoL have shown that measured with scales that include motor impairment, LiS patients show lower QoL than healthy controls.[13] But measured using scales that do not include motor impairment, their QoL is not significantly altered.[13] Mild and moderate depression is more common in LiS patients than healthy controls.[13] It is common for patients to be more emotionally sensitive and experience involuntary crying or laughter after the onset of LiS, compared to before,[7] a known problem after injuries to the brainstem.[14] Early medical stabilization and early rehabilitation improves prognosis[4 10] and a correct and early diagnosis is essential to minimize suffering and enable proper care[6]. When caused by an ischemic stroke, early stroke treatment such as anticoagulation and treatment with tissue plasminogen activator – t-PA could enhance the possibilities of a greater recovery.[15 16]

Participation is an aspect of disability which is dependent on both personal and environmental factors, as shown in the ICF-International Classification of Functioning, Disability and Health,[17] which is why it becomes an important issue to discuss. Neither autonomy nor

For peer review only - http://bmjopen.bmj.com/site/about/guidelines.xhtml

participation is static; they are values that can differ, throughout life[18] which means it can be reduced in one area but still be high in others. This becomes relevant in the topic of LiS, when motor function is low but cognitive function is high.

Severely disabled persons report good QoL despite their serious conditions, which is referred to as the disability paradox.[19] Despite researchers' belief that the disability paradox was real, it was only when Ubel et al. investigated potential sources of error and explanations of this good QoL, they were able to conclude that it did indeed exist.[20] This emphasizes the importance of caution for physicians and significant others when forced to make important decisions for their patients or next of kin, and the importance of not easily assuming a low life satisfaction.

The incidence of LiS in Sweden is unknown. Prior to 2015, there was no ICD-code for LiS in ICD-10-SE, which means The National Board of Health and Welfare has no statistics on the syndrome. LiS is not reported in the national quality register for stroke care, Swedish Stroke Registry(Riks-stroke).[21] One registry in Sweden, WebRehab, offers the possibility to report level of consciousness and also report LiS.[22] All the rehabilitation medicine clinics in Sweden participate in this quality register. There is a European Network for LiS patients but Sweden is not represented.[23]

To our knowledge, LiS has never been researched in Sweden. Previous studies have suggested that rehabilitation in these patients could improve if care was centralized and given by a skilled, professional team[3] and that early, intensive rehabilitation improves prognosis.[10] Therefore, there is a need for further research exploring LiS in Sweden, assessing possibilities and needs of that patient population. The aim of this study was to explore and describe LiS in Sweden with the purpose of gaining a better understanding of the life situation for this group

For peer review only - http://bmjopen.bmj.com/site/about/guidelines.xhtml

of patients. Internationally the information on LiS is also sparse and in particular, the long term situation for them. This study will contribute with some more information regarding LiS.

Material and Methods

This study was performed with material from the registers WebRehab, medical charts, the Swedish Tax Agency's population register, and The National Board of Health and Welfare's registry on Cause of Death (part one) and from questionnaires and interviews (part two).

WebRehab is a National Quality Registry in Rehabilitation Medicine. Twenty-three rehabilitation units in Sweden contribute to the database, representing all 21 counties of Sweden. The rehabilitation units report data from the rehabilitation period, including admission and discharge, and from a 1-year follow-up.[22] WebRehab is certified at level 2 which is the second highest certification level.

Study population

Eligible for this study were persons registered in WebRehab between 2007 - 2014 for whom the level of consciousness[24] was reported. All were diagnosed with locked-in syndrome (assessed by a specialist in Rehabilitation medicine in collaboration with neurologist) and had been admitted for in-patient rehabilitation in specialized rehabilitation medicine clinics. Twelve persons were identified from eight different hospitals. One person was excluded after the validation process due to not meeting the inclusion criteria and one person due to not having a valid personal identity number. For details, see fig 1.

Procedure

For peer review only - http://bmjopen.bmj.com/site/about/guidelines.xhtml

For peer review only

The Regional Ethical Review Board in Gothenburg approved this study. Application number was 052-15 and was accepted 20150415.

In part one, data from medical charts were gathered for validation and quality control of a quality register. No informed consent from the study participants is required as per Swedish law. In part two, informed, written consent was obtained from all participants or their representative.

During part one data were collected from registers. The identity of the included study participants was obtained from WebRehab´s database. The Functional Independence Measure – FIM – measures level of disability and independence in two scales, one motor scale and one cognitive scale[25] and was analysed for description of ADL-functions and dependency.

To make certain that the data in WebRehab had been entered correctly (a validation), the medical charts were checked. To obtain charts, care units were contacted, first by letters, then reminders were sent by email and attempts were made to reach persons in charge by phone.

Data from the Swedish Tax Agency's population register were collected to investigate how many of the study persons were still alive and to obtain their addresses and contact information.

Data on cause of death and date of death were obtained from The National Board of Health and Welfare's registry on Cause of Death. Both the application and the communication following the application were written and handled by the first author.

During part two questionnaires and interviews were performed. An information letter along with the questionnaires was sent to all study persons still alive. If no response, a reminder was

For peer review only

sent by letter and thereafter the attempts to reach the study persons or proxy was by phone. If the study persons wanted to participate, they were asked to send in the questionnaires or contact the author.

Four study persons who agreed to participate were visited for a personal, structured interview. The interviews were conducted in the participants' homes and were recorded and transcribed. The first author performed all interviews. During the interviews, a proxy gave some answers or a personal assistant and the rest were given by the participant and translated by next of kin. After the interview, the participant confirmed that the information given was correct, both the information given in the interview and in the questionnaires. One study participant was not able to perform a personal interview but participated through a telephone interview with her representative

Questionnaires

The different questionnaires used were Stroke Impact Scale (SIS-v3.0)[26], RAND-36, Impact on Participation and Autonomy (IPA-E)[27] and EQ-5D briefly described below.

SIS-v3.0

To assess health status a Swedish version of the SIS-v3.0[28] was used. The SIS is developed for, validated and shown to be reliable for stroke patients.[26 29]

RAND-36

RAND-36 is a survey instrument that assesses health-related Quality of Life. The Swedish version of RAND-36 is a modern translation of The Short form Health Survey – SF-36, but it is similar enough to allow comparisons. It is reliable and valid for measures on HRQoL in stroke patients.[30]

IPA-E

For peer review only - http://bmjopen.bmj.com/site/about/guidelines.xhtml

IPA-E was used to assess and measure impact on participation and autonomy. The original version of IPA has good validity and reliability[31] IPA-E includes 5 domains, autonomy indoors, family role, autonomy outdoors, social life and relationships, and work and education.[27]

EQ-5D

To assess Health Related Quality of Life (HRQoL), a Swedish version of the questionnaire EQ-5D was used. EQ-5D is a standardized, validated health questionnaire developed by the EuroQol Group Association.[32] It is intended to be used for self-completion in postal surveys, interviews and clinical practice.[33] The EQ-5D is a valid and reliable measure of HRQoL after stroke.[33] EQ-5D assesses health in 5 dimensions, mobility, self-care, usual activities, pain/discomfort, and anxiety/depression.

Statistical methods

For statistical analyses, IBM® *SPSS Statistics 21* was used. Mainly descriptive statistics with mean and median values were used for the demographic data.

.

For peer review only

Results

Population characteristics

The characteristics of the study population are summarized in table 1.

Table 1 Characteristics of the study population

Study persons	n=10
Alive	*7*
Deceased	*3*
Cause of LiS	
Ischemic stroke	*7*
Haemorrhagic stroke	*3*
Classification of LiS at admittance for rehabilitation	
Classic	*6*
Incomplete	*4*
Previous CVD/VRF	6
Total in hospital stay Median (Range)	163 days (150-449)
Length of Stay in Rehab Median (Range)	151 days (63-289)
Age at onset Median (Range)	49 y (22-67)
Survival* Median (Range)	1.9 y (1.5-2.3)
Time since onset** Median (Range)	5.9 y (2.3-8.1)

** Deceased study persons only included*

*** Alive study persons only included*

LiS - Locked-in syndrome, CVD - Cardiovascular disease, VRF - Vascular Risk Factors

Out of ten study persons, there were three women and seven men, age at onset varied from 22 years to 67 years with a median of 49 years. In six of the cases of ischemic stroke, the underlying cause was a basilar thrombosis and the seventh case, a vertebral artery dissection. Six persons had cardiovascular disease or documented vascular risk factors. The most

For peer review only - http://bmjopen.bmj.com/site/about/guidelines.xhtml

For peer review only

common vascular risk factor was hypertension. The patient were admitted to intensive care and then at stroke units before the rehabilitation period. The length of stay in before rehab varied between the hospitals and was not always dependent on the medical needs of the patient. Nine of the study persons experienced respiratory complications during hospitalization.

No change in ADL-dependency in motor scale domains was seen in any of the study persons when measured with FIM at admission to rehab and discharge; all were completely dependent in all domains. However, social-cognitive part of FIM improved with rehabilitation since communication was improved (which has an impacted in these items).

Seven persons from the total population (n=10) were still alive at start of study and three persons were deceased. One person died during rehabilitation and the remaining two after the initial rehabilitation period, time from onset to date of death was 1.5, 1.9 and 2.3 years. The cause of death was pulmonary embolism, acute myocardial infarction, and acute vascular disorders of the intestine. In none of the cases was the cause of death reported as being the result of respiratory complication due to LiS.

For details of all study persons and of participants, see tables 2 and 3

For peer review only

Table 2. All study persons

	Study persons									
	2	3	4	5	6	7	8	9	10	11
Aetiology	Vascular [illegible]	Vascular [illegible]	Vascular (I)	Vascular [illegible]	Vascular (I)	Vascular (H)	Vascular (I)	Vascular (H)	Vascular (I)	Vascular [illegible]
Classification	Classic	Classic	Incomplete	Classic	Classic	Incomplete	Incomplete	Incomplete	Classic	Classic
Discharged to	Nursing home	Short-term care	Short-term care	Nursing home	Independent living with personal assistance	Independent living with personal assistance	Died during rehab	Independent living with personal assistance	Short-term care	Nursing home
Survival	[2]	1.9 y	[2]	2.3 y	[2]	[2]	1.6 y	[2]	[2]	[2]
Cause of death (ICD-10-SE)		Pulmonary embolism (I26.9)		Acute vascular disorders of the intestine (K55.0)			Acute myocardial infarction (I21.9)			
Present form of residency	Nursing home		Apartment with community-based support		Independent living with personal assistance	Independent living with personal assistance		Independent living with personal assistance	Independent living with personal assistance	Nursing home

[1] Full years, [2] Still alive

Abbreviations: H - Haemorrhagic, I – Ischemic

For peer review only - http://bmjopen.bmj.com/site/about/guidelines.xhtml

For peer review only

For peer review only - http://bmjopen.bmj.com/site/about/guidelines.xhtml

Table 3. Assistance to and communication type of participants

	Followed up patients			
	2	4	6	10
Time since LiS	8 years	7.5 years	5.5 years	7 years
Living arrangement	Lives in a nursing home/home with society-based support. Joint common areas.	Lives in an apartment with society-based support. Lives alone.	Lives in a house. Lives with family.	Lives in a house. Lives with family.
Personal assistance	No personal assistants. Gets assistance from staff.	External personal assistants + extra assistance from staff when needed night-time	External personal assistance + partner as paid assistant	External personal assistance + partner as paid assistant
Amount	Assistance from staff 168h/week	Assistance - 168 h/week<br>Dual staffing - 10.5 h/week<br>Total – 178.5 h/week	Single staffing - 168 h/week.	Assistance - 168 h/week<br>Dual staffing - 80.5 h/week<br>Total – 248.5 h/week
Communication	Alphabet board, blinking	Mainly alphabet board (blinking sometimes)	Oral, alphabet board (pointing)	Blinking, eye-tracking device
Nutrition	Percutaneous endoscopic gastrostomy (PEG)	Oral, pureed/semi-solid	Oral, solid diet	PEG, occasional treat by mouth
Locomotion	Electric wheelchair	Electric wheelchair	Electric/manual wheelchair. Can walk a few steps with support	Electric wheelchair

For peer review only

For peer review only - http://bmjopen.bmj.com/site/about/guidelines.xhtml

HRQoL and impact on participation

Results from the questionnaires are presented separately for each questionnaire. No mean scores were calculated due to the low number of participants in the follow-up. To put the participants' scores in perspective, values for reference populations are included in the tables. The main finding from the questionnaires is that, although values vary between the follow-up participants, higher scores were seen in cognitive and mental domains (e.g. SIS-v3.0 Memory and Emotion and RAND-36 Mental Health) than in physical domains (e.g. SIS-v3.0 Strength, RAND-36 Physical Functioning and EQ-5D Mobility).

Individual scores on the SIS-v3.0 are presented in table 4, on RAND-36 in table 5, on IPA-E in table 6 and on EQ-5D in table 7.

Table 4 Stroke Impact Scale Results

	Individual score				Reference*
**Domain**	**2**	**4**	**6**	**10**	Mean *(SD)*
Strength	25	37.5	25	0	71 *(26.9)*
Hand function	0	0	30	0	81.1 *(22.2)*
Mobility	0	0	19.4	5.6	77.6 *(17.4)*
ADL	0	12.5	15	20	85 *(21)*
Emotion	44.4	94.4	88.9	86.1	75 *(28.5)*
Memory	100	42.9	100	71.4	75.6 *(29.2)*
Communication	75	85.7	53.6	14.3	68.9 *(34.5)*
Social participation	12.5	31.25	78.1	25	70.3 *(27.5)*
Stroke recovery	20	50	100	10	68.4 *(25.5)*

* Swedish stroke population, assessed 12 months after stroke.[28]

For peer review only - http://bmjopen.bmj.com/site/about/guidelines.xhtml

Table 5 RAND-36 Results

Domain	Individual score				Reference (LiS)* Mean *(SD)*	Reference (Stroke)** Mean *(95% CI)*
	2	4	6	10		
Physical functioning	0	0	5	0	0 *(0)*	51.2 *(44.3-58.1)*
Role limitations due to physical health	0	0	100	0	59.4 *(32.6)*	14.7 *(3.3-26.1)*
Role limitations due to emotional problems	33.3	100	100	100	75.0 *(34.5)*	18.0 *(10.2-25.8)*
Vitality/Energy-fatigue	45	55	75	70	64.4 *(24.6)*	42.9 *(37.8-48.0)*
Mental health/Emotional well-being	60	76	92	76	68 *(19.6)*	62.7 *(58.2-67.2)*
Social functioning	12.5	75	50	87.5	56.3 *(34.1)*	55.2 *(49.2-61.4)*
Bodily pain	80	90	100	67.5	82 *(26.8)*	65.0 *(57.9-72.1)*
General health	40	30	100	60	63.5 *(33.0)*	58.2 *(52.5-63.9)*

* Belgian LiS population, assessed more than 12 months after onset.[11]
** Swedish stroke population, assessed 2 years after day hospital rehabilitation for stroke.[34]

Table 6 Impact on Participation and Autonomy Questionnaire Results

Domain	Individual score				Reference * Mean *(SD)*
	2	4	6	10	
Autonomy indoors	4	3	0	2	0.96 *(0.6)*
Family role	4	4	4	4	1.96 *(1.1)*
Autonomy outdoors	4	3	2	3	2.35 *(0.9)*
Social life and relations	3	2	3	3	1.48 *(0.7)*
Work and education	-[1]	-[1]	1	-[1]	-

[1]Cannot be assessed, participant is not currently employed.
*Iranian stroke population, assessed 5-36 months after their stroke.[35]

For peer review only

Table 7 EuroQol-5 dimensions Results

Domain	7ndividual score				Reference* Mean *(95% CI)*
	2	4	6	10	
Mobility	3	3	3	3	-
Self-care	3	3	3	3	-
Usual activities	3	3	1	3	-
Pain/discomfort	3	2	1	2	-
Anxiety/depression	2	2	1	2	-
Index value	-0.429	-0.166	0.122	-0.166	0.44 *(0.28-0.42)*
VAS	0.02	0.5	1	0.4	62.7 *(58.8-66.6)*

*Swedish stroke population, assessed 2 years after day hospital rehabilitation for stroke.[34]

Interviews

Information

In all but one interview, the follow-up participants described a lack of information about LiS and it was difficult to get information about technical devices, or the follow-up procedure. They mentioned that they were sometimes forced to find technical aids and organize follow-up on their own. One participant, and a proxy, intended to write a book for persons with LiS about how to get information and support. This participant also found this information and support by becoming a member of a patient association.

When the follow-up participants were in intensive care, they felt they got better information than after discharge, although medical terms were sometimes difficult to understand.

Technical devices, technical adjustments and shortcomings

All used one or more wheel-chair/s; alphabet boards and used or wanted to use an eye-tracking computer device. This kind of computer is expensive and was not available for everyone.

For peer review only - http://bmjopen.bmj.com/site/about/guidelines.xhtml

An alarm button which can be placed by the temple, adjusted hospital beds and lifts, tilting table, ceiling hoist, a walking frame with extra support and a sit-to stand and transfer device were also mentioned as technical devices that the follow-up participants had access to. Two used bicycles for passive cycling. One person wanted a Functional Electric Stimulation/FES-assisted training device, but the municipality refused funding. This person was also part of a customer test group for a device, which combines eye-tracking technology with an electric wheelchair to enable control of the wheelchair with eye movements. He was promised money to purchase a wheelchair because of his help in developing the new technology.

Adjustments of homes were made such as installing ramps, widening doors, thresholds and elevated toilet seats.

Progress after rehabilitation and care

Several years after stroke, the follow-up participants' motor functions were still improving. They could communicate with the help of an alphabet board and used an eye-tracking computer device, or communicated mainly by blinking. One person had some oral communication including most vowels, some consonants and a few short words, today he can control his jaw and tongue muscles and he has some movement in his fingers. Another could walk a few steps with support, lift his arms and move his head; he also had some function in his left hand, but was still completely dependent in ADL. Two persons could eat solid food and had no additional nutritional support. One person had an alarm button with a pre-recorded sentence, which he could press by turning his head to attract attention. During the last year, he had learnt to shake his head and is currently practicing nodding. All needed a lot of daily support and had personal assistants around the clock and sometimes two assistants at the same time.

For peer review only - http://bmjopen.bmj.com/site/about/guidelines.xhtml

Likes and dislikes about care and authorities

The interviewed persons with LIS appreciated having phenomenal physiotherapists. Every second week both an occupational therapist and a physiotherapist visited one participant, she still wanted a lot more additional training on top of these visits. Some said contacts with authorities mostly worked well, although it could be time-consuming, but one argued that daily contacts with caregivers worked less well. One participant felt unhappy because she was in a nursing home and could not live on her own terms but was granted a flat of her own in due time.

Some follow-up participants/ proxies were offered support by a counsellor and were pleased with this. One partner talked about the difficult mourning process after LiS. Nothing turned out the way she had wanted because of her husband´s LiS. She was helped to think and reflect in a new way, like what could be planned and done despite LiS. One partner reflected that during intense rehabilitation periods, one gets a lot of support from all specialists but then it ends, for example appointments with a counsellor. One person wanted to meet a specialist doctor but felt he was not heard, having so many questions not answered by the local doctor.

Attitudes from professionals

All interviewed experienced not being treated as adults, people talked to them slowly and loudly as if they were not able to understand anything which was frustrating. This could be aggravating if a person treating you did not know you well enough and/or did not have enough knowledge about the syndrome. A proxy said that just being able to nod your head led to a participant being treated with more respect.

For peer review only - http://bmjopen.bmj.com/site/about/guidelines.xhtml

Family and friends

One participant had separated from her husband and had a complicated life situation. The others had mostly good family relationships, in two cases the wives worked as personal assistants. It was articulated that a relationship with a daughter had worsened after LiS and that this was the worst thing that had happened. Friends might have disappeared after LiS but the remaining ones were appreciated.

Quality of life

All but one evaluated their current life situation. They mentioned feeling joy at being alive and having the ability to encourage others. Improving quality of life could mean being granted more hours with personal assistants or getting in contact with a person with the same syndrome.

Discussion

Findings

We identified ten persons who had been diagnosed with LiS between 2007 and 2014 and investigated factors at onset, during the rehabilitation period, and after discharge. Seven of these persons were still alive at the start of the study and four of these participated. The common cause was stroke and most of these were ischemic. According to medical charts, some of the study persons improved in their motor function and all are still improving, although not in independency measured with the FIM motor scale. No major cognitive deficits were reported. Mortality in this population was 30 % and mean survival time for the deceased was 1.9 years. For the study persons still alive, mean time since onset of LiS was 5.9 years. From the interviews, we identified areas with unfulfilled needs: information, respect,

For peer review only - http://bmjopen.bmj.com/site/about/guidelines.xhtml

and specialized technical aids. The data from the questionnaires were mostly in line with information from the interviews, those who expressed high QoL and high impact on participation and autonomy in the interview, also scored high in domains not affected by motor impairments in the questionnaires.

Strengths and limitations

Since this was a nation-wide study and no selection was done, the number of patients might be considered few compared to previous studies abroad with study samples of around 20-30 persons.[5 13 36] One hypothesis could be that incidence numbers on LiS differ for some reason in Sweden. Another could be that incidence numbers are similar but not all persons with LiS in Sweden have been identified. This study population was identified through a register for patients having been admitted to rehabilitation medicine units and therefore, did not include patients who died in intensive care units. Up until the end of 2014, WebRehab was, to our knowledge, the only register in Sweden that had statistics on LiS but as of 1st of January 2015, there is an ICD-code in ICD-10-SE. What this will mean for the care of LiS-patients is hard to predict but reporting LiS will be possible for every care unit.

The questionnaires used are similar to each other in many ways, but all of them include unique aspects when compared to each other. EQ-5D is a quite rough instrument and physical functions have a large impact on the result. The value of using it in this particular study, where physical functions are very low, might therefore be worth discussing. In the EQ-5D, an index score under 0 is described as indicating a condition worse than death which is a problematic statement, e.g. three of our follow-up participants had an index score under 0, but none of them described their situation as worse than death.

When presenting the results of the questionnaires, values from reference populations are included in the tables, and our reasoning was to put our participants' scores in perspective. However, due to the low numbers of follow-up participants, no further comparisons can be

For peer review only - http://bmjopen.bmj.com/site/about/guidelines.xhtml

made. The reference groups are all stroke populations, because all our participants had suffered a stroke and are included in this patient group.

The method chosen for this study was a quantitative, descriptive method containing analysis of register data, medical charts and structured interviews based on questionnaires. The reason we chose this approach instead of a qualitative method with unstructured interviews was the follow-up participants' limited ability to communicate effectively. This we believe would mean unstructured interviews would not give more information than structured interviews. By combining questionnaires with personal interviews, the follow-up participants had a chance to elaborate on their answers and opinions while still maintaining a standardized form of assessment with the questionnaires.

Since a proxy or a personal assistant was present and involved in the interviews, there is a risk of misinterpretations in translations or that things were not relayed in the way the participant intended. All follow-up participants were therefore asked if the information given was correct at the end of the interviews.

Comparison with existing literature and guidelines

It seems consistent with previous knowledge that mortality is high during the first years after onset but when stabilized, persons with LiS may live for decades.[7] Since onset of LiS for our study population only goes back to 2007, real long term survival cannot be commented upon here.

The cause of death of the study persons was never a result of respiratory complications or problems with breathing, despite the high prevalence of respiratory complications during hospitalization. This might be explained by proper care, which has prevented or successfully treated any respiratory problems, but it might also be explained by simple luck.

The importance of maintaining vital functions and that fatality in LiS has declined with improvements in quality of medicine has been discussed in previous studies.[6] In line with

For peer review only - http://bmjopen.bmj.com/site/about/guidelines.xhtml

previous findings,[7 10 11] most of the study persons were middle-aged with a median age of 49 years at onset. In line with our study, patients with chronic LiS have slim chances of major improvements in motor function.[10]

According to FIM, five persons improved in independency in cognitive domains. Expression and problem solving are the two areas with the least improvements, which might be explained by the poor communication skills of this patient group.

In many aspects, the results on the questionnaires varied between the participants but most of them scored high on domains measuring emotional and cognitive functions and low on physical domains and functions. This is in line with previous knowledge that QoL in persons with LiS can be high scoring in mental domains, while low scoring in physical domains.[13]

Clinical and policy implications

Problems like nursing staff using complicated medical terminology or not getting enough information about technical and rehabilitation aids were brought up. As this patient group has limited communication skills, it seems important to fulfil all their needs for technical equipment to improve their QoL. All participants had experienced that they were not being treated with respect, and felt that people talked over their heads. These problems have been discussed in context of disability and aphasia, it affects social participation and with that, QoL in a negative way.[37 38] Raising awareness about LiS, both among caregivers and the public is necessary, since it might lead to less insecurity and incertitude when meeting a person with LiS and, therefore result in people treating a person with LiS with more respect.

Future research

From 2015, there will be an ICD-code in ICD-10-SE, which will improve the possibility of longitudinal research of this group of patients in Sweden. This seems to be an urgent field of

For peer review only - http://bmjopen.bmj.com/site/about/guidelines.xhtml

research because, to our knowledge, there are no articles yet from Sweden about LiS. In addition, improving the development and research of new technical devices for this group of patients might help them to communicate more efficiently and to find methods of improving motor functions.

Author contribution

KS, ÅN and KSS contributed to the design of the study. KS met the participants and gave them questionnaires, conducted and summarized the structured interviews. MT categorized the interviews inductively and wrote the manuscript together with KS and KSS.

Data sharing statement

Due to ethical restrictions, data are available upon request. Interested researchers may submit requests for data to the authors (contact Professor Katharina S. Sunnerhagen, email: ks.sunnerhagen@neuro.gu.se). According to Swedish regulation: http://www.epn.se/en/start/regulations/, the permission to use data is only for what has been applied for and then approved by the Ethical board. Not following the regulations is seen as scientific misconduct.

Competing interests

All authors declare that there is no conflict of interest.

Acknowledgements

We would like to acknowledge the help from WebRehab and the clinics that provided information on the persons. At finally yet importantly, we would like to thank the participants for answering questionnaires. Sincere thanks to them and their families for allowing KS to visit them and for generously telling about their situation and experiences. We would like to thank Dr Kate Bramley-Moore for her English language assistance and proofreading.

For peer review only

Funding

The study is in part funded by The Swedish Research Council (2017-00946) and a grant from the Swedish Heart and Lung Foundation (20160526). The supporting bodies had no influence on the content of the study.

Patient and Public Involvement

In the steering committee for the national quality register WebRehab, there are two representatives from patient organizations. There were no patient representatives involved in the recruitment to or conduction of the study. A short summary of the results has been distributed to the follow-up participants in the interviews.

For peer review only

References

1. Posner JB, Saper CB, Schiff ND, et al. Plum and Posner's Diagnosis of Stupor and Coma. 4 ed. New York: Oxford University Press 2007.
2. Patterson JR, Grabois M. Locked-in syndrome: a review of 139 cases. *Stroke* 1986;17(4):758-64. [published Online First: 1986/07/01]
3. Schjolberg A, Sunnerhagen KS. Unlocking the locked in; a need for team approach in rehabilitation of survivors with locked-in syndrome. *Acta Neurol Scand* 2012;125(3):192-8. doi: 10.1111/j.1600-0404.2011.01552.x [doi] [published Online First: 2011/06/29]
4. Doble JE, Haig AJ, Anderson C, et al. Impairment, activity, participation, life satisfaction, and survival in persons with locked-in syndrome for over a decade: follow-up on a previously reported cohort. *J Head Trauma Rehabil* 2003;18(5):435-44. [published Online First: 2003/09/16]
5. Katz RT, Haig AJ, Clark BB, et al. Long-term survival, prognosis, and life-care planning for 29 patients with chronic locked-in syndrome. *Arch Phys Med Rehabil* 1992;73(5):403-8. doi: 0003-9993(92)90025-R [pii] [published Online First: 1992/05/01]
6. Nikic PM, Jovanovic D, Paspalj D, et al. Clinical characteristics and outcome in the acute phase of ischemic locked-in syndrome: case series of twenty patients with ischemic LIS. *Eur Neurol* 2013;69(4):207-12. doi: 000345272 [pii]
10.1159/000345272 [doi] [published Online First: 2013/01/12]
7. Leon-Carrion J, van Eeckhout P, Dominguez-Morales Mdel R. The locked-in syndrome: a syndrome looking for a therapy. *Brain Inj* 2002;16(7):555-69. doi: 10.1080/02699050110119466 [doi] [published Online First: 2002/07/18]
8. American Congress of Rehabilitation M. Recommendations for use of uniform nomenclature pertinent to patients with severe alterations in consciousness. *Archives of Physical Medicine and Rehabilitation* 1995;76(2):205-09.
9. Pearce JM. The locked in syndrome. *Br Med J (Clin Res Ed)* 1987;294(6566):198-9. [published Online First: 1987/01/24]
10. Casanova E, Lazzari RE, Lotta S, et al. Locked-in syndrome: improvement in the prognosis after an early intensive multidisciplinary rehabilitation. *Arch Phys Med Rehabil* 2003;84(6):862-7. doi: S000399930300008X [pii] [published Online First: 2003/06/17]
11. Snoeys L, Vanhoof G, Manders E. Living with locked-in syndrome: an explorative study on health care situation, communication and quality of life. *Disabil Rehabil* 2013;35(9):713-8. doi: 10.3109/09638288.2012.705950 [doi] [published Online First: 2012/08/18]
12. Soderholm S, Meinander M, Alaranta H. Augmentative and alternative communication methods in locked-in syndrome. *J Rehabil Med* 2001;33(5):235-9. [published Online First: 2001/10/05]
13. Rousseau MC, Pietra S, Nadji M, et al. Evaluation of quality of life in complete locked-in syndrome patients. *Journal of Palliative Medicine* 2013;16(11):1455-58.
14. Parvizi J, Anderson SW, Martin CO, et al. Pathological laughter and crying: A link to the cerebellum. *Brain* 2001;124(9):1708-19.
15. Barbic D, Levine Z, Tampieri D, et al. Locked-in syndrome: a critical and time-dependent diagnosis. *CJEM* 2012;14(5):317-20. [published Online First: 2012/09/13]
16. Janjua N, Wartenberg KE, Meyers PM, et al. Reversal of locked-in syndrome with anticoagulation, induced hypertension, and intravenous t-PA. *Neurocrit Care* 2005;2(3):296-9. doi: NCC:2:3:296 [pii]

For peer review only - http://bmjopen.bmj.com/site/about/guidelines.xhtml

10.1385/NCC:2:3:296 [doi] [published Online First: 2005/09/15]
17. World Health Organization. International Classification of Functioning, Disability and Health (ICF). Geneva, 2001.
18. Cardol M, De Jong BA, Ward CD. On autonomy and participation in rehabilitation. *Disability and Rehabilitation* 2002;24(18):970-74.
19. Albrecht GL, Devlieger PJ. The disability paradox: High quality of life against all odds. *Social Science and Medicine* 1999;48(8):977-88.
20. Ubel PA, Schwarz N, Loewenstein G, et al. Misimagining the unimaginable: The disability paradox and health care decision making. *Health Psychology* 2005;24(4 SUPPL.):S57-S62.
21. Asplund K, Hulter Asberg K, Appelros P, et al. The Riks-Stroke story: building a sustainable national register for quality assessment of stroke care. *Int J Stroke* 2011;6(2):99-108. doi: 10.1111/j.1747-4949.2010.00557.x [doi] [published Online First: 2011/03/05]
22. Sunnerhagen KS, Flansbjer UB, Lannsjo M, et al. WebRehab: a Swedish database for quality control in rehabilitation. *J Rehabil Med* 2014;46(10):958-62. doi: 10.2340/16501977-1886 [doi] [published Online First: 2014/10/10]
23. Association du Locked in Syndrome ALIS. LISEF LIS European Foundation [Internet]. [cited 2015 26th May]. Available from: http://www.alis-asso.fr/lisef/ accessed May 26th 2015.
24. Giacino JT, Ashwal S, Childs N, et al. The minimally conscious state: Definition and diagnostic criteria. *Neurology* 2002;58(3):349-53.
25. Grimby G, Gudjonsson G, Rodhe M, et al. The functional independence measure in Sweden: experience for outcome measurement in rehabilitation medicine. *Scand J Rehabil Med* 1996;28(2):51-62. [published Online First: 1996/05/01]
26. Duncan PW, Wallace D, Lai SM, et al. The stroke impact scale version 2.0. Evaluation of reliability, validity, and sensitivity to change. *Stroke* 1999;30(10):2131-40. [published Online First: 1999/10/08]
27. Kersten P, Cardol M, George S, et al. Validity of the impact on participation and autonomy questionnaire: a comparison between two countries. *Disabil Rehabil* 2007;29(19):1502-9. doi: 771341328 [pii]
10.1080/09638280601030066 [doi] [published Online First: 2007/09/14]
28. Guidetti S, Ytterberg C, Ekstam L, et al. Changes in the impact of stroke between 3 and 12 months post-stroke, assessed with the Stroke Impact Scale. *J Rehabil Med* 2014;46(10):963-8. doi: 10.2340/16501977-1865 [doi] [published Online First: 2014/09/05]
29. Duncan PW, Bode RK, Min Lai S, et al. Rasch analysis of a new stroke-specific outcome scale: the Stroke Impact Scale. *Arch Phys Med Rehabil* 2003;84(7):950-63. doi: S0003999303000352 [pii] [published Online First: 2003/07/26]
30. Anderson C, Laubscher S, Burns R. Validation of the Short Form 36 (SF-36) health survey questionnaire among stroke patients. *Stroke* 1996;27(10):1812-16.
31. Larsson Lund M, Nordlund A, Nygard L, et al. Perceptions of participation and predictors of perceived problems with participation in persons with spinal cord injury. *J Rehabil Med* 2005;37(1):3-8. doi: W4GD12KB4ELWXC0T [pii]
10.1080/16501970410031246 [doi] [published Online First: 2005/03/25]
32. EuroQol Research Foundation. [Internet]. Rotterdam: EuroQol Research Foundation; 2015 [Available from: http://www.euroqol.org/about-eq-5d.html accessed Feb 6 2015.
33. Dorman PJ, Waddell F, Slattery J, et al. Is the EuroQol a valid measure of health-related quality of life after stroke? *Stroke* 1997;28(10):1876-82. [published Online First: 1997/10/28]

For peer review only - http://bmjopen.bmj.com/site/about/guidelines.xhtml

34. Grenthe Olsson B, Sunnerhagen KS. Functional and cognitive capacity and health-related quality of life 2 years after day hospital rehabilitation for stroke: a prospective study. *J Stroke Cerebrovasc Dis* 2007;16(5):208-15. doi: S1052-3057(07)00079-1 [pii]
10.1016/j.jstrokecerebrovasdis.2007.06.002 [doi] [published Online First: 2007/09/12]
35. Fallahpour M, Tham K, Joghataei MT, et al. Perceived participation and autonomy: aspects of functioning and contextual factors predicting participation after stroke. *J Rehabil Med* 2011;43(5):388-97. doi: 10.2340/16501977-0789 [doi] [published Online First: 2011/03/31]
36. Leon-Carrion J, van Eeckhout P, Dominguez-Morales Mdel R, et al. The locked-in syndrome: a syndrome looking for a therapy. *Brain Inj* 2002;16(7):571-82. doi: 10.1080/02699050110119781 [doi] [published Online First: 2002/07/18]
37. Dalemans RJ, de Witte L, Wade D, et al. Social participation through the eyes of people with aphasia. *Int J Lang Commun Disord* 2010;45(5):537-50. doi: 10.3109/13682820903223633 [pii]
10.3109/13682820903223633 [doi] [published Online First: 2009/10/21]
38. Wagner M. "Does he take sugar?". *World Health Forum* 1991;12(1):87-9. [published Online First: 1991/01/01]

For peer review only

Figure 1 legend

Flow chart of the study. One person was excluded after the validation process due to not meeting the inclusion criteria (locked in syndrome) and one person due to not having a valid personal identity number.

For peer review only

For peer review only - http://bmjopen.bmj.com/site/about/guidelines.xhtml

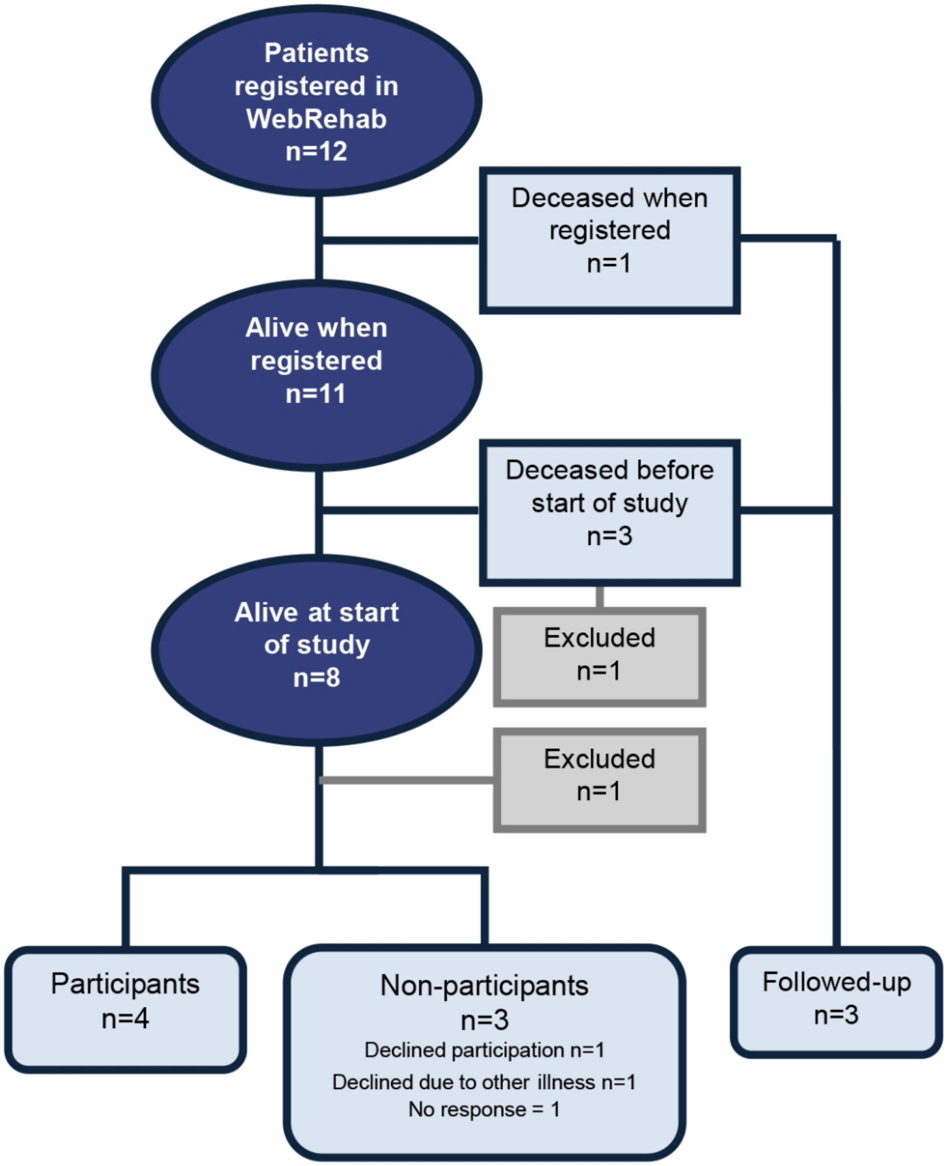

99x119mm (300 x 300 DPI)

STROBE 2007 (v4) Statement—Checklist of items that should be included in reports of *cohort studies*

Section/Topic	Item #	Recommendation	Reported on page #
**Title and abstract**	1	(*a*) Indicate the study's design with a commonly used term in the title or the abstract	1
		(*b*) Provide in the abstract an informative and balanced summary of what was done and what was found	2
**Introduction**			
Background/rationale	2	Explain the scientific background and rationale for the investigation being reported	4,5
Objectives	3	State specific objectives, including any prespecified hypotheses	6
**Methods**			
Study design	4	Present key elements of study design early in the paper	6
Setting	5	Describe the setting, locations, and relevant dates, including periods of recruitment, exposure, follow-up, and data collection	6,7
Participants	6	(*a*) Give the eligibility criteria, and the sources and methods of selection of participants. Describe methods of follow-up	7
		(*b*) For matched studies, give matching criteria and number of exposed and unexposed	NA
Variables	7	Clearly define all outcomes, exposures, predictors, potential confounders, and effect modifiers. Give diagnostic criteria, if applicable6	8.9
Data sources/ measurement	8*	For each variable of interest, give sources of data and details of methods of assessment (measurement). Describe comparability of assessment methods if there is more than one group	*8,9*
Bias	9	Describe any efforts to address potential sources of bias	NA
Study size	10	Explain how the study size was arrived at	8,9
Quantitative variables	11	Explain how quantitative variables were handled in the analyses. If applicable, describe which groupings were chosen and why	9
Statistical methods	12	(*a*) Describe all statistical methods, including those used to control for confounding	9
		(*b*) Describe any methods used to examine subgroups and interactions	NA
		(*c*) Explain how missing data were addressed	NA
		(*d*) If applicable, explain how loss to follow-up was addressed	NA
		(*e*) Describe any sensitivity analyses	NA
**Results**			

For peer review only

Participants	13*	(a) Report numbers of individuals at each stage of study—eg numbers potentially eligible, examined for eligibility, confirmed eligible, included in the study, completing follow-up, and analysed	9
		(b) Give reasons for non-participation at each stage	9
		(c) Consider use of a flow diagram	Fig 1
Descriptive data	14*	(a) Give characteristics of study participants (eg demographic, clinical, social) and information on exposures and potential confounders	10,12
		(b) Indicate number of participants with missing data for each variable of interest	10,12
		(c) Summarise follow-up time (eg, average and total amount)	
Outcome data	15*	Report numbers of outcome events or summary measures over time	12, 14.15,16
Main results	16	(*a*) Give unadjusted estimates and, if applicable, confounder-adjusted estimates and their precision (eg, 95% confidence interval). Make clear which confounders were adjusted for and why they were included	
		(*b*) Report category boundaries when continuous variables were categorized	
		(*c*) If relevant, consider translating estimates of relative risk into absolute risk for a meaningful time period	
Other analyses	17	Report other analyses done—eg analyses of subgroups and interactions, and sensitivity analyses	16,17
**Discussion**			
Key results	18	Summarise key results with reference to study objectives	20
**Limitations**			
Interpretation	20	Give a cautious overall interpretation of results considering objectives, limitations, multiplicity of analyses, results from similar studies, and other relevant evidence	20
Generalisability	21	Discuss the generalisability (external validity) of the study results	20
**Other information**			
Funding	22	Give the source of funding and the role of the funders for the present study and, if applicable, for the original study on which the present article is based	24

*Give information separately for cases and controls in case-control studies and, if applicable, for exposed and unexposed groups in cohort and cross-sectional studies.

Note: An Explanation and Elaboration article discusses each checklist item and gives methodological background and published examples of transparent reporting. The STROBE checklist is best used in conjunction with this article (freely available on the Web sites of PLoS Medicine at http://www.plosmedicine.org/, Annals of Internal Medicine at http://www.annals.org/, and Epidemiology at http://www.epidem.com/). Information on the STROBE Initiative is available at www.strobe-statement.org.

For peer review only

BMJ Open

Locked-in syndrome in Sweden an explorative study of persons who underwent rehabilitation; a cohort study

Journal:	*BMJ Open*
Manuscript ID	bmjopen-2018-023185.R2
Article Type:	Research
Date Submitted by the Author:	22-Jan-2019

Complete List of Authors:	Svernling, Kajsa; Univ of Gothenburg, Inst of Neuroscience and Physiology<br>Törnbom, Marie; Univ of Gothenburg, Inst of Neuroscience and Physiology<br>Nordin, Åsa; Univ of Gothenburg, Inst of Neuroscience and Physiology<br>Sunnerhagen, Katharina; Univ of Gothenburg, Inst of Neuroscience and Physiology

**Primary Subject Heading**:	Neurology
Secondary Subject Heading:	Rehabilitation medicine
Keywords:	Locked-in syndrome, Living situation, Quality of Life

SCHOLARONE™ Manuscripts

Locked-in syndrome in Sweden an explorative study of persons who underwent rehabilitation; a cohort study

Kajsa Svernling[1], MD, Marie Törnbom[1] , PhD medical social worker, Åsa Nordin[1] RPT, M Sc, Katharina S Sunnerhagen[1*], MD, PhD

[1]Institute of neuroscience and physiology, department of clinical neuroscience,

Sahlgrenska Academy, University of Gothenburg, Gothenburg, Sweden

*Corresponding author:

Katharina S Sunnerhagen

Email address: ks.sunnerhagen@neuro.gu.se

Telephone: +46 709520023

Postal address:

Rehabilitation medicine

Per Dubbsgatan 14, 3rd floor

Sahlgrenska University Hospital

S-413 45 Gothenburg

Sweden

Word count Abstract: 263

Word count Main manuscript: 3.935

Number of tables: 7

Number of figures: 1

For peer review only

Abstract

Objective: Locked-in syndrome (LiS) is a rare condition, characterized by a complete paresis except for vertical eye movements and blinking with cognitive functions intact, commonly caused by ischemia in the ventral pons. Previous studies have indicated that persons with LiS can live on for many years and have a good quality of life (QoL). To our knowledge, LiS has never been studied in Sweden. The aim was to explore LiS in Sweden; describing population characteristics, living situation, mortality/cause of death and health-related quality of life/impact on participation.

Design: Explorative, nation-wide study with two parts (quantitative and qualitative). Persons registered in the national quality register WebRehab during 2007-2014 were eligible.

Participants: Ten persons were identified in part one, four participated in part two. During part one data were collected from WebRehab, medical charts and registers, while questionnaires and interviews were used during part two.

Results: Seven out of ten were men, median age at onset was 49 years and the cause of LiS was in all cases stroke, 70% of which ischemic. Three were deceased with a median time of survival of 1.9 years. Seven were still alive, with a median time elapsed since onset of 5.8 years. Three participants experienced good quality of life. Information, respect from professionals, and more specialized technical devices were three areas containing unfulfilled needs.

Conclusion: This was the first study conducted in Sweden and the characteristics of this population were like those studied abroad. In this study, the persons with LiS that were interviewed expressed the need for proper care, appropriate technical aids and a supportive environment in order to have quality of life. However, there is still much room for improvements.

For peer review only

Key words: Locked-in syndrome; Living situation; Quality of Life

Strengths and limitations of this study (bullet points)

- The focus of this study was to explore and describe LiS, which has to our knowledge never been studied in Sweden. The study population constituted the whole population of patients with LiS receiving in-patient rehabilitation between 2007 and 2014.
- We managed to interview four participants of the study population with help from others, and this contributed substantially to the contents of the study.
- This study is limited by its small number of patients and the results can only be indicative and cannot be generalized. Worth noting is that most studies are fewer than ten or even single cases and as such, this study does not stand out as small.
- This study was performed in the Swedish context and the results relative to other cultural contexts need consideration.

For peer review only

Introduction

Locked-in Syndrome (LiS) is a state in which a patient is both quadriplegic and paralyzed up to the lower cranial nerves but conscious and with retained control of vertical eye movement and eyelids. LiS is usually caused by a brain stem lesion, most commonly a ventral pontine lesion that interrupts the descending pyramidal tracts.[1-3] The lesion is often a result of an ischemic stroke due to thrombosis in the basilar artery, but can also be caused by haemorrhages, trauma, tumours or ischemia due to hypotension.[4-6] In rare cases, the cause is metabolic or infectious.[7]

The American Congress of Rehabilitation Medicine[8] recommends neurobehavioral criteria to be used when diagnosing LiS. The criteria are 1: Eye opening well sustained, 2: Basic cognitive abilities preserved (clinical examination), 3: Severe hypophonia or aphonia on clinical examination, 4: Quadriplegia/Quadriparesis on clinical examination and 5: Communication primarily through eye movements or through blinking. The LiS can vary in clinical presentation. *Classic LiS* is defined as a fully paralyzed patient with intact vertical eye movements and movement in the eyelid. *Incomplete LiS* is similar to Classic LiS but with remnants of motor functions beyond those of the classic variant, usually some movement in fingers and/or toes. *Total LiS* is defined as total immobility, the use of electroencephalography – EEG is then necessary to ascertain consciousness. Regarding duration, LiS can be chronic or transient. In the latter, the patients recover so that they not anymore fulfil the criteria for LIS, but still might have (often substantial) sequelae due to their stroke.

The view on prognosis of LiS has shifted a lot through the years. When LiS first became a subject of study, the opinion was that acute mortality was high,[2] with nearly no long-term

For peer review only - http://bmjopen.bmj.com/site/about/guidelines.xhtml

survivors.[9] Since then opinion has shifted, numbers on mortality still vary between studies but the overall view on survival is more positive. If the patient medically stabilizes and survives the first year, 5-year survival may be up to 81-86 %,[5 10] and some patients survive for decades.[7]

Patients with chronic LiS often continue to have highly impaired motor function, even if some improvement is possible.[3 10 11] Among other things, the impairments lead to them becoming dependent in the Activities of Daily Living – ADL (self-care etc.). Tetraplegia, along with impairments in breathing patterns, also means that respiratory complications are common.[3] Most patients living with LiS learn to communicate in some way.[7 10 12]

Studies on quality of life – QoL have shown that measured with scales that include motor impairment, LiS patients show lower QoL than healthy controls.[13] But measured using scales that do not include motor impairment, their QoL is not significantly altered.[13] Mild and moderate depression is more common in LiS patients than in healthy controls.[13] It is common for patients to be more emotionally sensitive and experience involuntary crying or laughter after the onset of LiS, compared to before,[7] a known problem after injuries to the brainstem.[14] Early medical stabilization and early rehabilitation improves prognosis[4 10] and a correct and early diagnosis is essential to minimize suffering and enable proper care[6]. When caused by an ischemic stroke, early stroke treatment such as anticoagulation and treatment with tissue plasminogen activator (t-PA) could enhance the possibilities of a greater recovery.[15 16]

Participation is an aspect of disability which is dependent on both personal and environmental factors, as shown in the ICF-International Classification of Functioning, Disability and Health,[17] which is why it becomes an important issue to discuss. Neither autonomy nor participation is static; they are values that can differ throughout life[18], which means it can be

For peer review only

reduced in one area but still be high in others. This becomes relevant in the topic of LiS, when motor function is low but cognitive function is high.

Severely disabled persons report good QoL despite their serious conditions, which is referred to as the disability paradox.[19] Despite researchers' belief that the disability paradox was real, it was only when Ubel et al. investigated potential sources of error and explanations of this good QoL that they were able to conclude that it did indeed exist.[20] This emphasizes the importance of caution for physicians and significant others when forced to make important decisions for their patients or next of kin, and the importance of not easily assuming a low life satisfaction.

The incidence of LiS in Sweden is unknown. Prior to 2015, there was no ICD-code for LiS in ICD-10-SE, which means The National Board of Health and Welfare has no statistics on the syndrome. LiS is not reported in the national quality register for stroke care, Swedish Stroke Registry(Riks-stroke).[21] One registry in Sweden, WebRehab, offers the possibility to report level of consciousness and also report LiS.[22] All the rehabilitation medicine clinics in Sweden participate in this quality register. There is a European Network for LiS patients but Sweden is not represented.[23]

To our knowledge, LiS has never been researched in Sweden. Previous studies have suggested that rehabilitation in these patients could improve if care was centralized and given by a skilled, professional team[3] and that early, intensive rehabilitation improves prognosis.[10] Therefore, there is a need for further research exploring LiS in Sweden, assessing possibilities and needs of that patient population. The aim of this study was to explore and describe LiS in Sweden with the purpose of gaining a better understanding of the life situation for this group of patients. Internationally the information on LiS is also sparse, and in particular the long term situation for them. This study will contribute some more information regarding LiS.

For peer review only - http://bmjopen.bmj.com/site/about/guidelines.xhtml

Material and Methods

This study was performed with material from the registers WebRehab, medical charts, the Swedish Tax Agency's population register, and The National Board of Health and Welfare's registry on Cause of Death (part one) and from questionnaires and interviews (part two).

WebRehab is a National Quality Registry in Rehabilitation Medicine. Twenty-three rehabilitation units in Sweden contribute to the database, representing all 21 counties of Sweden. The rehabilitation units report data from the rehabilitation period, including admission and discharge, and from a 1-year follow-up.[22] WebRehab is certified at level 2 which is the second highest certification level.

Study population

Eligible for this study were persons registered in WebRehab between 2007 and 2014 for whom the level of consciousness[24] was reported. All were diagnosed with LiS (assessed by a specialist in Rehabilitation medicine in collaboration with neurologist) and had been admitted for in-patient rehabilitation in specialized rehabilitation medicine clinics. In WebRehab, the following is stated as for locked-in syndrome: "the patient has tetraplegia and cannot use speech to communicate but is fully preserved cognitively, there is retained eye opening please exclude bilateral ptos that may complicate the condition), aphonia or hypophonia, tetraplegia or tetraparesis. There is also a question regarding communication (adequate yes/no using vertical or horizontal eye movements alternatively eye blinking

For peer review only - http://bmjopen.bmj.com/site/about/guidelines.xhtml

Twelve persons were identified from eight different hospitals. One person was excluded after the validation process due to not meeting the inclusion criteria and one person due to not having a valid personal identity number. For details, see fig 1.

Procedure

The Regional Ethical Review Board in Gothenburg approved this study. The application was assigned number 052-15 and was accepted 20150415.

In part one, data from medical charts were gathered for validation and quality control of a quality register. No informed consent from the study participants is required as per Swedish law. In part two, informed, written consent was obtained from all participants or their representative.

During part one data were collected from registers. The identity of the included study participants was obtained from WebRehab´s database. The Functional Independence Measure – FIM – measures level of disability and independence in two scales, one motor scale and one cognitive scale[25] and was analysed for description of ADL-functions and dependency.

To make certain that the data in WebRehab had been entered correctly (a validation), the medical charts were checked. To obtain charts, care units were contacted, first by letter, then reminders were sent by email, and finally attempts were made to reach persons in charge by phone.

For peer review only

Data from the Swedish Tax Agency's population register were collected to investigate how many of the study persons were still alive and to obtain their addresses and contact information.

Data on cause of death and date of death were obtained from The National Board of Health and Welfare's registry on Cause of Death. Both the application and the communication following the application were written and handled by the first author.

During part two questionnaires and interviews were performed. An information letter along with the questionnaires was sent to all study persons still alive. If no response, a reminder was sent by letter and thereafter the attempts to reach the study persons or proxy was by phone. If the study persons wanted to participate, they were asked to send in the questionnaires or contact the author.

Four study persons who agreed to participate were visited for a personal, structured interview. The interviews were conducted in the participants' homes and were recorded and transcribed. The first author performed all interviews. During the interviews, a proxy gave some answers or a personal assistant and the rest were given by the participant and translated by next of kin. After the interview, the participant confirmed that the information given was correct, both the information given in the interview and in the questionnaires. One study participant was not able to perform a personal interview but participated through a telephone interview with her representative.

Questionnaires

The different questionnaires used were Stroke Impact Scale (SIS-v3.0)[26], RAND-36, Impact on Participation and Autonomy (IPA-E)[27] and EQ-5D briefly described below.

For peer review only

SIS-v3.0

To assess health status a Swedish version of the SIS-v3.0[28] was used. The SIS is developed for, validated, and shown to be reliable for stroke patients.[26 29]

RAND-36

RAND-36 is a survey instrument that assesses health-related Quality of Life. The Swedish version of RAND-36 is a modern translation of The Short form Health Survey – SF-36, but it is similar enough to allow comparisons. It is reliable and valid for measures on HRQoL in stroke patients.[30]

IPA-E

IPA-E was used to assess and measure impact on participation and autonomy. The original version of IPA has good validity and reliability[31] IPA-E includes 5 domains: autonomy indoors, family role, autonomy outdoors, social life and relationships, and work and education.[27]

EQ-5D

To assess Health Related Quality of Life (HRQoL), a Swedish version of the questionnaire EQ-5D was used. EQ-5D is a standardized, validated health questionnaire developed by the EuroQol Group Association.[32] It is intended to be used for self-completion in postal surveys, interviews and clinical practice.[33] The EQ-5D is a valid and reliable measure of HRQoL after stroke.[33] EQ-5D assesses health in 5 dimensions: mobility, self-care, usual activities, pain/discomfort, and anxiety/depression.

Statistical methods

For statistical analyses, IBM® *SPSS Statistics 21* was used. Mainly descriptive statistics with mean and median values were used for the demographic data.

For peer review only - http://bmjopen.bmj.com/site/about/guidelines.xhtml

For peer review only

Results

Population characteristics

The characteristics of the study population are summarized in table 1.

Table 1 Characteristics of the study population

Study persons	n=10
Alive	*7*
Deceased	*3*
Cause of LiS	
Ischemic stroke	*7*
Haemorrhagic stroke	*3*
Classification of LiS at admittance for rehabilitation	
Classic	*6*
Incomplete	*4*
Previous CVD/VRF	6
Total in hospital stay Median (Range)	216 days (150-627)
Length of Stay in Rehab Median (Range)	151 days (63-345)
Age at onset Median (Range)	49 y (22-67)
Survival* Median (Range)	1.9 y (1.5-2.3)
Time since onset** Median (Range)	5.9 y (2.3-8.1)

** Deceased study persons only included*

*** Alive study persons only included*

LiS - Locked-in syndrome, CVD - Cardiovascular disease, VRF - Vascular Risk Factors

Out of ten study persons, there were three women and seven men; age at onset varied from 22 years to 67 years with a median of 49 years. In six of the cases of ischemic stroke, the underlying cause was a basilar thrombosis, and the seventh case a vertebral artery dissection. Six persons had cardiovascular disease or documented vascular risk factors. The most

For peer review only - http://bmjopen.bmj.com/site/about/guidelines.xhtml

common vascular risk factor was hypertension. The patients were admitted to intensive care and then at stroke units before the rehabilitation period. The length of stay in hospital? before rehab varied between the hospitals and was not always dependent on the medical needs of the patient. Nine of the study persons experienced respiratory complications during hospitalization.

No change in ADL-dependency in motor scale domains was seen in any of the study persons when measured with FIM at admission to rehab and discharge; all were completely dependent in all domains. However, the social-cognitive part of FIM improved with rehabilitation since communication was improved (which has an impact in these items).

Seven persons from the total population (n=10) were still alive at start of study and three persons were deceased. One person died during rehabilitation and the remaining two after the initial rehabilitation period, time from onset to date of death was 1.5, 1.9 and 2.3 years. The cause of death was pulmonary embolism, acute myocardial infarction, and acute vascular disorders of the intestine. In none of the cases was the cause of death reported as being the result of respiratory complication due to LiS.

For details of all study persons and of participants, see tables 2 and 3

For peer review only

Table 2. All study persons

	Study persons									
	2	3	4	5	6	7	8	9	10	11
Aetiology	Vascular [illegible]	Vascular [illegible]	Vascular (I)	Vascular [illegible]	Vascular (I)	Vascular (H)	Vascular (I)	Vascular (H)	Vascular (I)	Vascular [illegible]
Classification	Classic	Classic	Incomplete	Classic	Classic	Incomplete	Incomplete	Incomplete	Classic	Classic
Discharged to	Nursing home	Short-term care	Short-term care	Nursing home	Independent living with personal assistance	Independent living with personal assistance	Died during rehab	Independent living with personal assistance	Short-term care	Nursing home
Survival	1	1.9 y	1	2.3 y	1	1	1.6 y	1	1	1
Cause of death (ICD-10-SE)		Pulmonary embolism (I26.9)		Acute vascular disorders of the intestine (K55.0)			Acute myocardial infarction (I21.9)			
Present form of residency	Nursing home		Apartment with community-based support		Independent living with personal assistance	Independent living with personal assistance		Independent living with personal assistance	Independent living with personal assistance	Nursing home

1 Still alive

Abbreviations: H - Haemorrhagic, I – Ischemic

For peer review only - http://bmjopen.bmj.com/site/about/guidelines.xhtml

For peer review only

For peer review only - http://bmjopen.bmj.com/site/about/guidelines.xhtml

Table 3. Assistance to and communication type of participants

	Followed up patients			
	2	4	6	10
Time since LiS	8 years	7.5 years	5.5 years	7 years
Living arrangement	Lives in a nursing home/home with society-based support. Joint common areas.	Lives in an apartment with society-based support. Lives alone.	Lives in a house. Lives with family.	Lives in a house. Lives with family.
Personal assistance	No personal assistants. Gets assistance from staff.	External personal assistants + extra assistance from staff when needed night-time	External personal assistance + partner as paid assistant	External personal assistance + partner as paid assistant
Amount	Assistance from staff 168h/week	Assistance - 168 h/week<br>Dual staffing - 10.5 h/week<br>Total – 178.5 h/week	Single staffing - 168 h/week.	Assistance - 168 h/week<br>Dual staffing - 80.5 h/week<br>Total – 248.5 h/week
Communication	Alphabet board, blinking	Mainly alphabet board (blinking sometimes)	Oral, alphabet board (pointing)	Blinking, eye-tracking device
Nutrition	Percutaneous endoscopic gastrostomy (PEG)	Oral, pureed/semi-solid	Oral, solid diet	PEG, occasional treat by mouth
Locomotion	Electric wheelchair	Electric wheelchair	Electric/manual wheelchair. Can walk a few steps with support	Electric wheelchair

For peer review only

HRQoL and impact on participation

Results from the questionnaires are presented separately for each questionnaire. No mean scores were calculated due to the low number of participants in the follow-up. To put the participants' scores in perspective, values for reference populations are included in the tables. The main finding from the questionnaires is that, although values vary between the follow-up participants, higher scores were seen in cognitive and mental domains (e.g. SIS-v3.0 Memory and Emotion and RAND-36 Mental Health) than in physical domains (e.g. SIS-v3.0 Strength, RAND-36 Physical Functioning and EQ-5D Mobility).

Individual scores on the SIS-v3.0 are presented in table 4, on RAND-36 in table 5, on IPA-E in table 6 and on EQ-5D in table 7.

Table 4 Stroke Impact Scale Results

	**Individual score**				**Reference*** Mean *(SD)*
**Domain**	**2**	**4**	**6**	**10**	
Strength	25	37.5	25	0	71 *(26.9)*
Hand function	0	0	30	0	81.1 *(22.2)*
Mobility	0	0	19.4	5.6	77.6 *(17.4)*
ADL	0	12.5	15	20	85 *(21)*
Emotion	44.4	94.4	88.9	86.1	75 *(28.5)*
Memory	100	42.9	100	71.4	75.6 *(29.2)*
Communication	75	85.7	53.6	14.3	68.9 *(34.5)*
Social participation	12.5	31.25	78.1	25	70.3 *(27.5)*
Stroke recovery	20	50	100	10	68.4 *(25.5)*

* Swedish stroke population, assessed 12 months after stroke.[28]

For peer review only - http://bmjopen.bmj.com/site/about/guidelines.xhtml

Table 5 RAND-36 Results

Domain	Individual score 2	4	6	10	Reference (LiS)* Mean *(SD)*	Reference (Stroke)** Mean *(95% CI)*
Physical functioning	0	0	5	0	0 *(0)*	51.2 *(44.3-58.1)*
Role limitations due to physical health	0	0	100	0	59.4 *(32.6)*	14.7 *(3.3-26.1)*
Role limitations due to emotional problems	33.3	100	100	100	75.0 *(34.5)*	18.0 *(10.2-25.8)*
Vitality/Energy-fatigue	45	55	75	70	64.4 *(24.6)*	42.9 *(37.8-48.0)*
Mental health/Emotional well-being	60	76	92	76	68 *(19.6)*	62.7 *(58.2-67.2)*
Social functioning	12.5	75	50	87.5	56.3 *(34.1)*	55.2 *(49.2-61.4)*
Bodily pain	80	90	100	67.5	82 *(26.8)*	65.0 *(57.9-72.1)*
General health	40	30	100	60	63.5 *(33.0)*	58.2 *(52.5-63.9)*

* Belgian LiS population, assessed more than 12 months after onset.[11]
** Swedish stroke population, assessed 2 years after day hospital rehabilitation for stroke.[34]

Table 6 Impact on Participation and Autonomy Questionnaire Results

Domain	Individual score 2	4	6	10	Reference * Mean *(SD)*
Autonomy indoors	4	3	0	2	0.96 *(0.6)*
Family role	4	4	4	4	1.96 *(1.1)*
Autonomy outdoors	4	3	2	3	2.35 *(0.9)*
Social life and relations	3	2	3	3	1.48 *(0.7)*
Work and education	-[1]	-[1]	1	-[1]	-

[1]Cannot be assessed, participant is not currently employed.
*Iranian stroke population, assessed 5-36 months after their stroke.[35]

For peer review only

For peer review only - http://bmjopen.bmj.com/site/about/guidelines.xhtml

Table 7 EuroQol-5 dimensions Results

Domain	Individual score 2	4	6	10	Reference* Mean *(95% CI)*
Mobility	3	3	3	3	-
Self-care	3	3	3	3	-
Usual activities	3	3	1	3	-
Pain/discomfort	3	2	1	2	-
Anxiety/depression	2	2	1	2	-
Index value	-0.429	-0.166	0.122	-0.166	0.44 *(0.38-0.50)*
VAS	0.02	0.5	1	0.4	62.7 *(58.8-66.6)*

*Swedish stroke population, assessed 2 years after day hospital rehabilitation for stroke.[34]

Interviews

Information

In all but one interview, the follow-up participants described a lack of information about LiS and it was difficult to get information about technical devices, or the follow-up procedure. They mentioned that they were sometimes forced to find technical aids and organize follow-up on their own. One participant, and a proxy, intended to write a book for persons with LiS about how to get information and support. This participant also found this information and support by becoming a member of a patient association.

When the follow-up participants were in intensive care, they felt they got better information than after discharge, although medical terms were sometimes difficult to understand.

Technical devices, technical adjustments and shortcomings

All used one or more wheel-chair/s; alphabet boards and used or wanted to use an eye-tracking computer device. This kind of computer is expensive and was not available for everyone.

For peer review only - http://bmjopen.bmj.com/site/about/guidelines.xhtml

An alarm button which can be placed by the temple, adjusted hospital beds and lifts, tilting table, ceiling hoist, a walking frame with extra support and a sit-to stand and transfer device were also mentioned as technical devices that the follow-up participants had access to. Two used bicycles for passive cycling. One person wanted a Functional Electric Stimulation/FES-assisted training device, but the municipality refused funding. This person was also part of a customer test group for a device which combines eye-tracking technology with an electric wheelchair to enable control of the wheelchair with eye movements. He was promised money to purchase a wheelchair because of his help in developing the new technology.

Adjustments of homes were made, such as installing ramps, widening doors, thresholds and elevated toilet seats.

Progress after rehabilitation and care

Several years after stroke, the follow-up participants' motor functions were still improving. They could communicate with the help of an alphabet board and used an eye-tracking computer device, or communicated mainly by blinking. One person had some oral communication including most vowels, some consonants and a few short words at discharge. At the time of assessment, he could control his jaw and tongue muscles and he had some movement in his fingers. Another could walk a few steps with support, lift his arms and move his head; he also had some function in his left hand, but was still completely dependent in ADL. Two persons could eat solid food and had no additional nutritional support. One person had an alarm button with a pre-recorded sentence, which he could press by turning his head to attract attention. During the last year, he had learnt to shake his head and is currently practicing nodding. All needed a lot of daily support and had personal assistants around the clock and sometimes two assistants at the same time.

For peer review only - http://bmjopen.bmj.com/site/about/guidelines.xhtml

Likes and dislikes about care and authorities

The interviewed persons with LIS appreciated having phenomenal physiotherapists during the rehabilitation period. Every second week both an occupational therapist and a physiotherapist visited one participant, she still wanted a lot more additional training on top of these visits. Some said contacts with authorities mostly worked well, although it could be time-consuming, but one argued that daily contacts with caregivers worked less well. One participant felt unhappy because she was in a nursing home and could not live on her own terms but was granted a flat of her own in due time.

Some follow-up participants/ proxies were offered support by a counsellor and were pleased with this. One partner talked about the difficult mourning process after LiS. Nothing turned out the way she had wanted because of her husband´s LiS. She was helped to think and reflect in a new way, such as what could be planned and done despite LiS. One partner reflected that during intense rehabilitation periods, one gets a lot of support from all specialists but then it ends, for example appointments with a counsellor. One person wanted to meet a specialist doctor but felt he was not heard, having so many questions not answered by the local doctor.

Attitudes from professionals

All interviewed experienced not being treated as adults, people talked to them slowly and loudly as if they were not able to understand anything which was frustrating. This could be aggravating if a person treating you did not know you well enough and/or did not have enough knowledge about the syndrome. A proxy said that just being able to nod your head led to a participant being treated with more respect.

For peer review only - http://bmjopen.bmj.com/site/about/guidelines.xhtml

Family and friends

One participant had separated from her husband and had a complicated life situation. The others had mostly good family relationships, in two cases the wives worked as personal assistants. It was articulated that a relationship with a daughter had worsened after LiS and that this was the worst thing that had happened. Friends might have disappeared after LiS but the remaining ones were appreciated.

Quality of life

All but one evaluated their current life situation. They mentioned feeling joy at being alive and having the ability to encourage others. Improving quality of life could mean being granted more hours with personal assistants or getting in contact with a person with the same syndrome.

Discussion

Findings

We identified ten persons who had been diagnosed with LiS between 2007 and 2014 and investigated factors at onset, during the rehabilitation period, and after discharge. Seven of these persons were still alive at the start of the study and four of these participated. The common cause was stroke and most of these were ischemic. According to medical charts, some of the study persons improved in their motor function and all are still improving, although not in independency measured with the FIM motor scale. No major cognitive deficits were reported. Mortality in this population was 30 % and mean survival time for the deceased was 1.9 years. For the study persons still alive, mean time since onset of LiS was 5.9 years. From the interviews, we identified areas with unfulfilled needs: information, respect,

For peer review only - http://bmjopen.bmj.com/site/about/guidelines.xhtml

For peer review only

and specialized technical aids. The data from the questionnaires were mostly in line with information from the interviews: those who expressed high QoL and high impact on participation and autonomy in the interview, also scored high in domains not affected by motor impairments in the questionnaires.

Strengths and limitations

Since this was a nation-wide study and no selection was done, the number of patients might be considered few compared to previous studies abroad with study samples of around 20-30 persons.[5 13 36] One hypothesis could be that incidence numbers on LiS differ for some reason in Sweden. Another could be that incidence numbers are similar but not all persons with LiS in Sweden have been identified. This study population was identified through a register for patients having been admitted to rehabilitation medicine units and therefore, did not include patients who died in intensive care units. Up until the end of 2014, WebRehab was, to our knowledge, the only register in Sweden that had statistics on LiS but as of 1st of January 2015, there is an ICD-code in ICD-10-SE. What this will mean for the care of LiS-patients is hard to predict but reporting LiS will be possible for every care unit.

The questionnaires used are similar to each other in many ways, but all of them include unique aspects when compared to each other. EQ-5D is a quite rough instrument and physical functions have a large impact on the result. The value of using it in this particular study, where physical functions are very low, might therefore be worth discussing. In the EQ-5D, an index score under 0 is described as indicating a condition worse than death which is a problematic statement. For instance, three of our follow-up participants had an index score under 0, but none of them described their situation as worse than death.

When presenting the results of the questionnaires, values from reference populations are included in the tables, and our reasoning was to put our participants' scores in perspective. However, due to the low numbers of follow-up participants, no further comparisons can be

For peer review only - http://bmjopen.bmj.com/site/about/guidelines.xhtml

made. The reference groups are all stroke populations, because all our participants had suffered a stroke and are included in this patient group.

The method chosen for this study was a quantitative, descriptive method containing analysis of register data, medical charts and structured interviews based on questionnaires. The reason we chose this approach instead of a qualitative method with unstructured interviews was the follow-up participants' limited ability to communicate effectively. This we believe would mean unstructured interviews would not give more information than structured interviews. By combining questionnaires with personal interviews, the follow-up participants had a chance to elaborate on their answers and opinions while still maintaining a standardized form of assessment with the questionnaires.

Since a proxy or a personal assistant was present and involved in the interviews, there is a risk of misinterpretations in translations or that things were not relayed in the way the participant intended. All follow-up participants were therefore asked if the information given was correct at the end of the interviews.

Comparison with existing literature and guidelines

It seems consistent with previous knowledge that mortality is high during the first years after onset but when stabilized, persons with LiS may live for decades.[7] Since onset of LiS for our study population only goes back to 2007, real long-term survival cannot be commented upon here.

The cause of death of the study persons was never a result of respiratory complications or problems with breathing, despite the high prevalence of respiratory complications during hospitalization. This might be explained by proper care, which has prevented or successfully treated any respiratory problems, but it might also be explained by chance.

The importance of maintaining vital functions and that fatality in LiS has declined with improvements in quality of medicine has been discussed in previous studies.[6] In line with

For peer review only - http://bmjopen.bmj.com/site/about/guidelines.xhtml

previous findings,[7 10 11] most of the study persons were middle-aged with a median age of 49 years at onset. In line with our study, patients with chronic LiS have slim chances of major improvements in motor function.[10]

According to FIM, five persons improved in independency in cognitive domains. Expression and problem solving are the two areas with the least improvements, which might be explained by the poor communication skills of this patient group.

In many aspects, the results on the questionnaires varied between the participants but most of them scored high on domains measuring emotional and cognitive functions and low on physical domains and functions. This is in line with previous knowledge that QoL in persons with LiS can be high scoring in mental domains, while low scoring in physical domains.[13]

Clinical and policy implications

Problems like nursing staff using complicated medical terminology or not getting enough information about technical and rehabilitation aids were brought up. As this patient group has limited communication skills, it seems important to fulfil all their needs for technical equipment to improve their QoL. All participants had experienced that they were not being treated with respect, and felt that people talked over their heads. These problems have been discussed in context of disability and aphasia, it affects social participation and with that, QoL in a negative way.[37 38] Raising awareness about LiS, both among caregivers and the public is necessary, since it might lead to less insecurity and incertitude when meeting a person with LiS and, therefore result in people treating a person with LiS with more respect.

Future research

From 2015, there will be an ICD-code in ICD-10-SE, which will improve the possibility of longitudinal research of this group of patients in Sweden. This seems to be an urgent field of

For peer review only - http://bmjopen.bmj.com/site/about/guidelines.xhtml

research because, to our knowledge, there are no articles yet from Sweden about LiS. In addition, improving the development and research of new technical devices for this group of patients might help them to communicate more efficiently and to find methods of improving motor functions.

Author contribution

KS, ÅN and KSS contributed to the design of the study. KS met the participants and gave them questionnaires, conducted and summarized the structured interviews. MT categorized the interviews inductively and wrote the manuscript together with KS and KSS.

Data sharing statement

Due to ethical restrictions, data are available upon request. Interested researchers may submit requests for data to the authors (contact Professor Katharina S. Sunnerhagen, email: ks.sunnerhagen@neuro.gu.se). According to Swedish regulation: http://www.epn.se/en/start/regulations/, the permission to use data is only for what has been applied for and then approved by the Ethical board. Not following the regulations is seen as scientific misconduct.

Competing interests

All authors declare that there is no conflict of interest.

Acknowledgements

We would like to acknowledge the help from WebRehab and the clinics that provided information on the persons. At finally yet importantly, we would like to thank the participants for answering questionnaires. Sincere thanks to them and their families for allowing KS to visit them and for generously telling about their situation and experiences. We would like to thank Dr Kate Bramley-Moore for her English language assistance and proofreading.

For peer review only - http://bmjopen.bmj.com/site/about/guidelines.xhtml

Funding

The study is in part funded by The Swedish Research Council (2017-00946) and a grant from the Swedish Heart and Lung Foundation (20160526). The supporting bodies had no influence on the content of the study.

Patient and Public Involvement

In the steering committee for the national quality register WebRehab, there are two representatives from patient organizations. There were no patient representatives involved in the recruitment to or conduction of the study. A short summary of the results has been distributed to the follow-up participants in the interviews.

For peer review only

For peer review only - http://bmjopen.bmj.com/site/about/guidelines.xhtml

References

1. Posner JB, Saper CB, Schiff ND, et al. Plum and Posner's Diagnosis of Stupor and Coma. 4 ed. New York: Oxford University Press 2007.
2. Patterson JR, Grabois M. Locked-in syndrome: a review of 139 cases. *Stroke* 1986;17(4):758-64. [published Online First: 1986/07/01]
3. Schjolberg A, Sunnerhagen KS. Unlocking the locked in; a need for team approach in rehabilitation of survivors with locked-in syndrome. *Acta Neurol Scand* 2012;125(3):192-8. doi: 10.1111/j.1600-0404.2011.01552.x [doi] [published Online First: 2011/06/29]
4. Doble JE, Haig AJ, Anderson C, et al. Impairment, activity, participation, life satisfaction, and survival in persons with locked-in syndrome for over a decade: follow-up on a previously reported cohort. *J Head Trauma Rehabil* 2003;18(5):435-44. [published Online First: 2003/09/16]
5. Katz RT, Haig AJ, Clark BB, et al. Long-term survival, prognosis, and life-care planning for 29 patients with chronic locked-in syndrome. *Arch Phys Med Rehabil* 1992;73(5):403-8. doi: 0003-9993(92)90025-R [pii] [published Online First: 1992/05/01]
6. Nikic PM, Jovanovic D, Paspalj D, et al. Clinical characteristics and outcome in the acute phase of ischemic locked-in syndrome: case series of twenty patients with ischemic LIS. *Eur Neurol* 2013;69(4):207-12. doi: 000345272 [pii]
10.1159/000345272 [doi] [published Online First: 2013/01/12]
7. Leon-Carrion J, van Eeckhout P, Dominguez-Morales Mdel R. The locked-in syndrome: a syndrome looking for a therapy. *Brain Inj* 2002;16(7):555-69. doi: 10.1080/02699050110119466 [doi] [published Online First: 2002/07/18]
8. American Congress of Rehabilitation M. Recommendations for use of uniform nomenclature pertinent to patients with severe alterations in consciousness. *Archives of Physical Medicine and Rehabilitation* 1995;76(2):205-09.
9. Pearce JM. The locked in syndrome. *Br Med J (Clin Res Ed)* 1987;294(6566):198-9. [published Online First: 1987/01/24]
10. Casanova E, Lazzari RE, Lotta S, et al. Locked-in syndrome: improvement in the prognosis after an early intensive multidisciplinary rehabilitation. *Arch Phys Med Rehabil* 2003;84(6):862-7. doi: S000399930300008X [pii] [published Online First: 2003/06/17]
11. Snoeys L, Vanhoof G, Manders E. Living with locked-in syndrome: an explorative study on health care situation, communication and quality of life. *Disabil Rehabil* 2013;35(9):713-8. doi: 10.3109/09638288.2012.705950 [doi] [published Online First: 2012/08/18]
12. Soderholm S, Meinander M, Alaranta H. Augmentative and alternative communication methods in locked-in syndrome. *J Rehabil Med* 2001;33(5):235-9. [published Online First: 2001/10/05]
13. Rousseau MC, Pietra S, Nadji M, et al. Evaluation of quality of life in complete locked-in syndrome patients. *Journal of Palliative Medicine* 2013;16(11):1455-58.
14. Parvizi J, Anderson SW, Martin CO, et al. Pathological laughter and crying: A link to the cerebellum. *Brain* 2001;124(9):1708-19.
15. Barbic D, Levine Z, Tampieri D, et al. Locked-in syndrome: a critical and time-dependent diagnosis. *CJEM* 2012;14(5):317-20. [published Online First: 2012/09/13]
16. Janjua N, Wartenberg KE, Meyers PM, et al. Reversal of locked-in syndrome with anticoagulation, induced hypertension, and intravenous t-PA. *Neurocrit Care* 2005;2(3):296-9. doi: NCC:2:3:296 [pii]

For peer review only - http://bmjopen.bmj.com/site/about/guidelines.xhtml

10.1385/NCC:2:3:296 [doi] [published Online First: 2005/09/15]
17. World Health Organization. International Classification of Functioning, Disability and Health (ICF). Geneva, 2001.
18. Cardol M, De Jong BA, Ward CD. On autonomy and participation in rehabilitation. *Disability and Rehabilitation* 2002;24(18):970-74.
19. Albrecht GL, Devlieger PJ. The disability paradox: High quality of life against all odds. *Social Science and Medicine* 1999;48(8):977-88.
20. Ubel PA, Schwarz N, Loewenstein G, et al. Misimagining the unimaginable: The disability paradox and health care decision making. *Health Psychology* 2005;24(4 SUPPL.):S57-S62.
21. Asplund K, Hulter Asberg K, Appelros P, et al. The Riks-Stroke story: building a sustainable national register for quality assessment of stroke care. *Int J Stroke* 2011;6(2):99-108. doi: 10.1111/j.1747-4949.2010.00557.x [doi] [published Online First: 2011/03/05]
22. Sunnerhagen KS, Flansbjer UB, Lannsjo M, et al. WebRehab: a Swedish database for quality control in rehabilitation. *J Rehabil Med* 2014;46(10):958-62. doi: 10.2340/16501977-1886 [doi] [published Online First: 2014/10/10]
23. Association du Locked in Syndrome ALIS. LISEF LIS European Foundation [Internet]. [cited 2015 26th May]. Available from: http://www.alis-asso.fr/lisef/ accessed May 26th 2015.
24. Giacino JT, Ashwal S, Childs N, et al. The minimally conscious state: Definition and diagnostic criteria. *Neurology* 2002;58(3):349-53.
25. Grimby G, Gudjonsson G, Rodhe M, et al. The functional independence measure in Sweden: experience for outcome measurement in rehabilitation medicine. *Scand J Rehabil Med* 1996;28(2):51-62. [published Online First: 1996/05/01]
26. Duncan PW, Wallace D, Lai SM, et al. The stroke impact scale version 2.0. Evaluation of reliability, validity, and sensitivity to change. *Stroke* 1999;30(10):2131-40. [published Online First: 1999/10/08]
27. Kersten P, Cardol M, George S, et al. Validity of the impact on participation and autonomy questionnaire: a comparison between two countries. *Disabil Rehabil* 2007;29(19):1502-9. doi: 771341328 [pii]
10.1080/09638280601030066 [doi] [published Online First: 2007/09/14]
28. Guidetti S, Ytterberg C, Ekstam L, et al. Changes in the impact of stroke between 3 and 12 months post-stroke, assessed with the Stroke Impact Scale. *J Rehabil Med* 2014;46(10):963-8. doi: 10.2340/16501977-1865 [doi] [published Online First: 2014/09/05]
29. Duncan PW, Bode RK, Min Lai S, et al. Rasch analysis of a new stroke-specific outcome scale: the Stroke Impact Scale. *Arch Phys Med Rehabil* 2003;84(7):950-63. doi: S0003999303000352 [pii] [published Online First: 2003/07/26]
30. Anderson C, Laubscher S, Burns R. Validation of the Short Form 36 (SF-36) health survey questionnaire among stroke patients. *Stroke* 1996;27(10):1812-16.
31. Larsson Lund M, Nordlund A, Nygard L, et al. Perceptions of participation and predictors of perceived problems with participation in persons with spinal cord injury. *J Rehabil Med* 2005;37(1):3-8. doi: W4GD12KB4ELWXC0T [pii]
10.1080/16501970410031246 [doi] [published Online First: 2005/03/25]
32. EuroQol Research Foundation. [Internet]. Rotterdam: EuroQol Research Foundation; 2015 [Available from: http://www.euroqol.org/about-eq-5d.html accessed Feb 6 2015.
33. Dorman PJ, Waddell F, Slattery J, et al. Is the EuroQol a valid measure of health-related quality of life after stroke? *Stroke* 1997;28(10):1876-82. [published Online First: 1997/10/28]

For peer review only - http://bmjopen.bmj.com/site/about/guidelines.xhtml

34. Grenthe Olsson B, Sunnerhagen KS. Functional and cognitive capacity and health-related quality of life 2 years after day hospital rehabilitation for stroke: a prospective study. *J Stroke Cerebrovasc Dis* 2007;16(5):208-15. doi: S1052-3057(07)00079-1 [pii]
10.1016/j.jstrokecerebrovasdis.2007.06.002 [doi] [published Online First: 2007/09/12]
35. Fallahpour M, Tham K, Joghataei MT, et al. Perceived participation and autonomy: aspects of functioning and contextual factors predicting participation after stroke. *J Rehabil Med* 2011;43(5):388-97. doi: 10.2340/16501977-0789 [doi] [published Online First: 2011/03/31]
36. Leon-Carrion J, van Eeckhout P, Dominguez-Morales Mdel R, et al. The locked-in syndrome: a syndrome looking for a therapy. *Brain Inj* 2002;16(7):571-82. doi: 10.1080/02699050110119781 [doi] [published Online First: 2002/07/18]
37. Dalemans RJ, de Witte L, Wade D, et al. Social participation through the eyes of people with aphasia. *Int J Lang Commun Disord* 2010;45(5):537-50. doi: 10.3109/13682820903223633 [pii]
10.3109/13682820903223633 [doi] [published Online First: 2009/10/21]
38. Wagner M. "Does he take sugar?". *World Health Forum* 1991;12(1):87-9. [published Online First: 1991/01/01]

For peer review only

For peer review only - http://bmjopen.bmj.com/site/about/guidelines.xhtml

Figure 1 legend

Flow chart of the study. One person was excluded after the validation process due to not meeting the inclusion criteria (locked in syndrome) and one person due to not having a valid personal identity number.

For peer review only

For peer review only - http://bmjopen.bmj.com/site/about/guidelines.xhtml

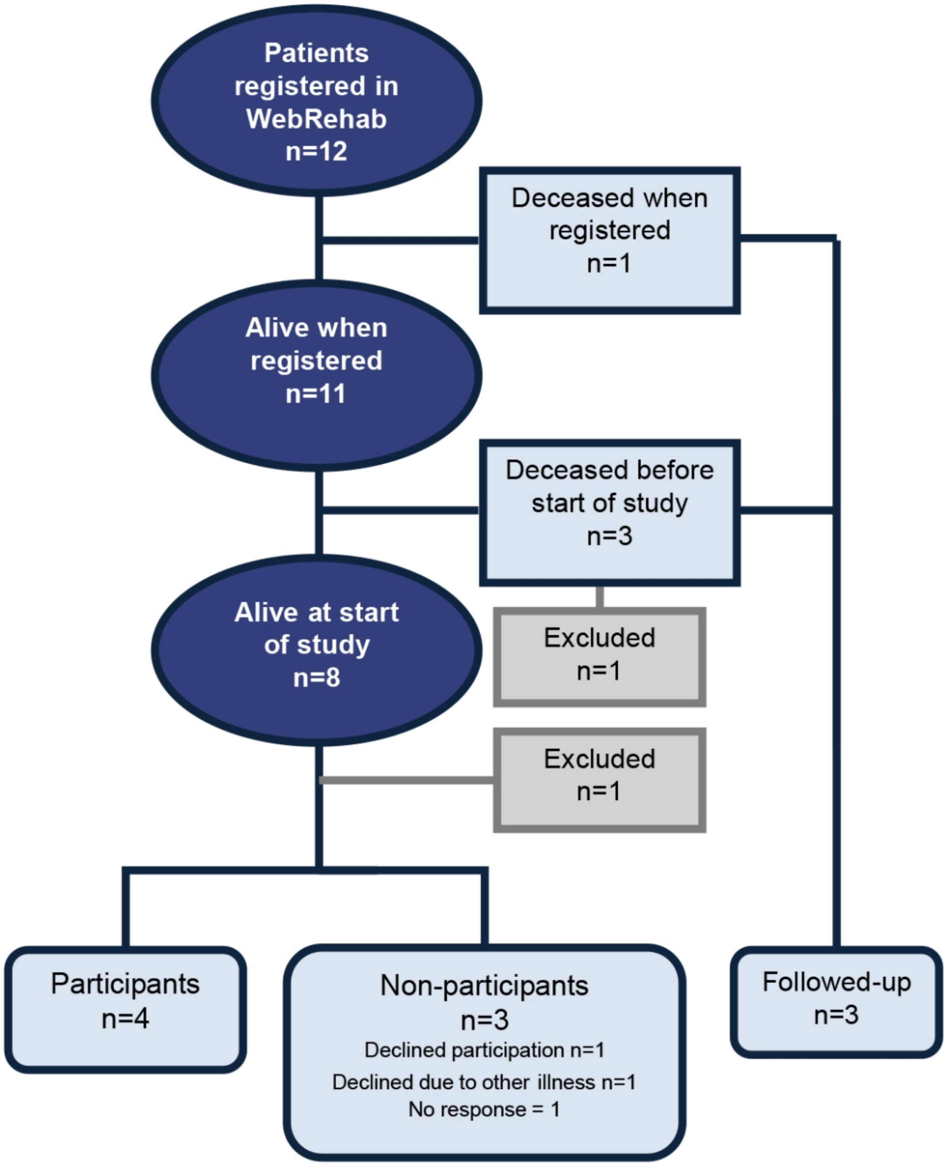

99x119mm (300 x 300 DPI)

STROBE 2007 (v4) Statement—Checklist of items that should be included in reports of *cohort studies*

Section/Topic	Item #	Recommendation	Reported on page #
**Title and abstract**	1	(*a*) Indicate the study's design with a commonly used term in the title or the abstract	1
		(*b*) Provide in the abstract an informative and balanced summary of what was done and what was found	2
**Introduction**			
Background/rationale	2	Explain the scientific background and rationale for the investigation being reported	4,5
Objectives	3	State specific objectives, including any prespecified hypotheses	6
**Methods**			
Study design	4	Present key elements of study design early in the paper	6
Setting	5	Describe the setting, locations, and relevant dates, including periods of recruitment, exposure, follow-up, and data collection	6,7
Participants	6	(*a*) Give the eligibility criteria, and the sources and methods of selection of participants. Describe methods of follow-up	7
		(*b*) For matched studies, give matching criteria and number of exposed and unexposed	NA
Variables	7	Clearly define all outcomes, exposures, predictors, potential confounders, and effect modifiers. Give diagnostic criteria, if applicable6	8.9
Data sources/ measurement	8*	For each variable of interest, give sources of data and details of methods of assessment (measurement). Describe comparability of assessment methods if there is more than one group	*8,9*
Bias	9	Describe any efforts to address potential sources of bias	NA
Study size	10	Explain how the study size was arrived at	8,9
Quantitative variables	11	Explain how quantitative variables were handled in the analyses. If applicable, describe which groupings were chosen and why	9
Statistical methods	12	(*a*) Describe all statistical methods, including those used to control for confounding	9
		(*b*) Describe any methods used to examine subgroups and interactions	NA
		(*c*) Explain how missing data were addressed	NA
		(*d*) If applicable, explain how loss to follow-up was addressed	NA
		(*e*) Describe any sensitivity analyses	NA
**Results**			

For peer review only

Participants	13*	(a) Report numbers of individuals at each stage of study—eg numbers potentially eligible, examined for eligibility, confirmed eligible, included in the study, completing follow-up, and analysed	9
		(b) Give reasons for non-participation at each stage	9
		(c) Consider use of a flow diagram	Fig 1
Descriptive data	14*	(a) Give characteristics of study participants (eg demographic, clinical, social) and information on exposures and potential confounders	10,12
		(b) Indicate number of participants with missing data for each variable of interest	10,12
		(c) Summarise follow-up time (eg, average and total amount)	
Outcome data	15*	Report numbers of outcome events or summary measures over time	12, 14.15,16
Main results	16	(*a*) Give unadjusted estimates and, if applicable, confounder-adjusted estimates and their precision (eg, 95% confidence interval). Make clear which confounders were adjusted for and why they were included	
		(*b*) Report category boundaries when continuous variables were categorized	
		(*c*) If relevant, consider translating estimates of relative risk into absolute risk for a meaningful time period	
Other analyses	17	Report other analyses done—eg analyses of subgroups and interactions, and sensitivity analyses	16,17
**Discussion**			
Key results	18	Summarise key results with reference to study objectives	20
**Limitations**			
Interpretation	20	Give a cautious overall interpretation of results considering objectives, limitations, multiplicity of analyses, results from similar studies, and other relevant evidence	20
Generalisability	21	Discuss the generalisability (external validity) of the study results	20
**Other information**			
Funding	22	Give the source of funding and the role of the funders for the present study and, if applicable, for the original study on which the present article is based	24

*Give information separately for cases and controls in case-control studies and, if applicable, for exposed and unexposed groups in cohort and cross-sectional studies.

Note: An Explanation and Elaboration article discusses each checklist item and gives methodological background and published examples of transparent reporting. The STROBE checklist is best used in conjunction with this article (freely available on the Web sites of PLoS Medicine at http://www.plosmedicine.org/, Annals of Internal Medicine at http://www.annals.org/, and Epidemiology at http://www.epidem.com/). Information on the STROBE Initiative is available at www.strobe-statement.org.

For peer review only

BMJ Open

Locked-in syndrome in Sweden, an explorative study of persons who underwent rehabilitation: a cohort study

Journal:	*BMJ Open*
Manuscript ID	bmjopen-2018-023185.R3
Article Type:	Research
Date Submitted by the Author:	15-Feb-2019
Complete List of Authors:	Svernling, Kajsa; Univ of Gothenburg, Inst of Neuroscience and Physiology<br>Törnbom, Marie; Univ of Gothenburg, Inst of Neuroscience and Physiology<br>Nordin, Åsa; Univ of Gothenburg, Inst of Neuroscience and Physiology<br>Sunnerhagen, Katharina; Univ of Gothenburg, Inst of Neuroscience and Physiology
**Primary Subject Heading**:	Neurology
Secondary Subject Heading:	Rehabilitation medicine
Keywords:	Locked-in syndrome, Living situation, Quality of Life

SCHOLARONE™
Manuscripts

Locked-in syndrome in Sweden, an explorative study of persons who underwent rehabilitation: a cohort study

Kajsa Svernling[1], MD, Marie Törnbom[1] , PhD medical social worker, Åsa Nordin[1] RPT, M Sc, Katharina S Sunnerhagen[1*], MD, PhD

[1]Institute of neuroscience and physiology, department of clinical neuroscience,

Sahlgrenska Academy, University of Gothenburg, Gothenburg, Sweden

*Corresponding author:

Katharina S Sunnerhagen

Email address: ks.sunnerhagen@neuro.gu.se

Telephone: +46 709520023

Postal address:

Rehabilitation medicine

Per Dubbsgatan 14, 3rd floor

Sahlgrenska University Hospital

S-413 45 Gothenburg

Sweden

Word count Abstract: 263

Word count Main manuscript: 3.935

Number of tables: 7

Number of figures: 1

For peer review only

Abstract

Objective: Locked-in syndrome (LiS) is a rare condition, characterized by a complete paresis except for vertical eye movements and blinking with cognitive functions intact, commonly caused by ischemia in the ventral pons. Previous studies have indicated that persons with LiS can live on for many years and have a good quality of life (QoL). To our knowledge, LiS has never been studied in Sweden. The aim was to explore LiS in Sweden; describing population characteristics, living situation, mortality/cause of death and health-related quality of life/impact on participation.

Design: Explorative, nation-wide study with two parts (quantitative and qualitative). Persons registered in the national quality register WebRehab during 2007-2014 were eligible.

Participants: Ten persons were identified in part one, four participated in part two. During part one data were collected from WebRehab, medical charts and registers, while questionnaires and interviews were used during part two.

Results: Seven out of ten were men, median age at onset was 49 years and the cause of LiS was in all cases stroke, 70% of which ischemic. Three were deceased with a median time of survival of 1.9 years. Seven were still alive, with a median time elapsed since onset of 5.8 years. Three participants experienced good quality of life. Information, respect from professionals, and more specialized technical devices were three areas containing unfulfilled needs.

Conclusion: This was the first study conducted in Sweden and the characteristics of this population were like those studied abroad. In this study, the persons with LiS that were interviewed expressed the need for proper care, appropriate technical aids and a supportive environment in order to have quality of life. However, there is still much room for improvements.

For peer review only

Key words: Locked-in syndrome; Living situation; Quality of Life

Strengths and limitations of this study (bullet points)

- The focus of this study was to explore and describe LiS, which has to our knowledge never been studied in Sweden. The study population constituted the whole population of patients with LiS receiving in-patient rehabilitation between 2007 and 2014.
- We managed to interview four participants of the study population with help from others, and this contributed substantially to the contents of the study.
- This study is limited by its small number of patients and the results can only be indicative and cannot be generalized. Worth noting is that most studies are fewer than ten or even single cases and as such, this study does not stand out as small.
- This study was performed in the Swedish context and the results relative to other cultural contexts need consideration.

For peer review only

Introduction

Locked-in Syndrome (LiS) is a state in which a patient is both quadriplegic and paralyzed up to the lower cranial nerves but conscious and with retained control of vertical eye movement and eyelids. LiS is usually caused by a brain stem lesion, most commonly a ventral pontine lesion that interrupts the descending pyramidal tracts.[1-3] The lesion is often a result of an ischemic stroke due to thrombosis in the basilar artery, but can also be caused by haemorrhages, trauma, tumours or ischemia due to hypotension.[4-6] In rare cases, the cause is metabolic or infectious.[7]

The American Congress of Rehabilitation Medicine[8] recommends neurobehavioral criteria to be used when diagnosing LiS. The criteria are 1: Eye opening well sustained, 2: Basic cognitive abilities preserved (clinical examination), 3: Severe hypophonia or aphonia on clinical examination, 4: Quadriplegia/Quadriparesis on clinical examination and 5: Communication primarily through eye movements or through blinking. The LiS can vary in clinical presentation. *Classic LiS* is defined as a fully paralyzed patient with intact vertical eye movements and movement in the eyelid. *Incomplete LiS* is similar to Classic LiS but with remnants of motor functions beyond those of the classic variant, usually some movement in fingers and/or toes. *Total LiS* is defined as total immobility, the use of electroencephalography – EEG is then necessary to ascertain consciousness. Regarding duration, LiS can be chronic or transient. In the latter, the patients recover so that they not anymore fulfil the criteria for LIS, but still might have (often substantial) sequelae due to their stroke.

The view on prognosis of LiS has shifted a lot through the years. When LiS first became a subject of study, the opinion was that acute mortality was high,[2] with nearly no long-term

For peer review only

survivors.[9] Since then opinion has shifted, numbers on mortality still vary between studies but the overall view on survival is more positive. If the patient medically stabilizes and survives the first year, 5-year survival may be up to 81-86 %,[5 10] and some patients survive for decades.[7]

Patients with chronic LiS often continue to have highly impaired motor function, even if some improvement is possible.[3 10 11] Among other things, the impairments lead to them becoming dependent in the Activities of Daily Living – ADL (self-care etc.). Tetraplegia, along with impairments in breathing patterns, also means that respiratory complications are common.[3] Most patients living with LiS learn to communicate in some way.[7 10 12]

Studies on quality of life – QoL have shown that measured with scales that include motor impairment, LiS patients show lower QoL than healthy controls.[13] But measured using scales that do not include motor impairment, their QoL is not significantly altered.[13] Mild and moderate depression is more common in LiS patients than in healthy controls.[13] It is common for patients to be more emotionally sensitive and experience involuntary crying or laughter after the onset of LiS, compared to before,[7] a known problem after injuries to the brainstem.[14] Early medical stabilization and early rehabilitation improves prognosis[4 10] and a correct and early diagnosis is essential to minimize suffering and enable proper care[6]. When caused by an ischemic stroke, early stroke treatment such as anticoagulation and treatment with tissue plasminogen activator (t-PA) could enhance the possibilities of a greater recovery.[15 16]

Participation is an aspect of disability which is dependent on both personal and environmental factors, as shown in the ICF-International Classification of Functioning, Disability and Health,[17] which is why it becomes an important issue to discuss. Neither autonomy nor participation is static; they are values that can differ throughout life[18], which means it can be

For peer review only

reduced in one area but still be high in others. This becomes relevant in the topic of LiS, when motor function is low but cognitive function is high.

Severely disabled persons report good QoL despite their serious conditions, which is referred to as the disability paradox.[19] Despite researchers' belief that the disability paradox was real, it was only when Ubel et al. investigated potential sources of error and explanations of this good QoL that they were able to conclude that it did indeed exist.[20] This emphasizes the importance of caution for physicians and significant others when forced to make important decisions for their patients or next of kin, and the importance of not easily assuming a low life satisfaction.

The incidence of LiS in Sweden is unknown. Prior to 2015, there was no ICD-code for LiS in ICD-10-SE, which means The National Board of Health and Welfare has no statistics on the syndrome. LiS is not reported in the national quality register for stroke care, Swedish Stroke Registry(Riks-stroke).[21] One registry in Sweden, WebRehab, offers the possibility to report level of consciousness and also report LiS.[22] All the rehabilitation medicine clinics in Sweden participate in this quality register. There is a European Network for LiS patients but Sweden is not represented.[23]

To our knowledge, LiS has never been researched in Sweden. Previous studies have suggested that rehabilitation in these patients could improve if care was centralized and given by a skilled, professional team[3] and that early, intensive rehabilitation improves prognosis.[10] Therefore, there is a need for further research exploring LiS in Sweden, assessing possibilities and needs of that patient population. The aim of this study was to explore and describe LiS in Sweden with the purpose of gaining a better understanding of the life situation for this group of patients. Internationally the information on LiS is also sparse, and in particular the long term situation for them. This study will contribute some more information regarding LiS.

For peer review only

Material and Methods

This study was performed with material from the registers WebRehab, medical charts, the Swedish Tax Agency's population register, and The National Board of Health and Welfare's registry on Cause of Death (part one) and from questionnaires and interviews (part two).

WebRehab is a National Quality Registry in Rehabilitation Medicine. Twenty-three rehabilitation units in Sweden contribute to the database, representing all 21 counties of Sweden. The rehabilitation units report data from the rehabilitation period, including admission and discharge, and from a 1-year follow-up.[22] WebRehab is certified at level 2 which is the second highest certification level.

Study population

Eligible for this study were persons registered in WebRehab between 2007 and 2014 for whom the level of consciousness[24] was reported. All were diagnosed with LiS (assessed by a specialist in Rehabilitation medicine in collaboration with neurologist) and had been admitted for in-patient rehabilitation in specialized rehabilitation medicine clinics. In WebRehab, the following is stated as for locked-in syndrome: "the patient has tetraplegia and cannot use speech to communicate but is fully preserved cognitively, there is retained eye opening please exclude bilateral ptos that may complicate the condition), aphonia or hypophonia, tetraplegia or tetraparesis. There is also a question regarding communication (adequate yes/no using vertical or horizontal eye movements alternatively eye blinking

For peer review only

Twelve persons were identified from eight different hospitals. One person was excluded after the validation process due to not meeting the inclusion criteria and one person due to not having a valid personal identity number. For details, see fig 1.

Procedure

The Regional Ethical Review Board in Gothenburg approved this study. The application was assigned number 052-15 and was accepted 20150415. There it is stated, that results will be used for a scientific publication as well as possible presentations at conferences. This is also clearly stated (as required by law) in the information letter to the presumptive study participants. They (or their next of kin) have given written informed consent, as well as oral, to publication.

In part one, data from medical charts were gathered for validation and quality control of a quality register. No informed consent from the study participants is required as per Swedish law. In part two, informed, written consent was obtained from all participants or their representative. This included consent for publication.

During part one data were collected from registers. The identity of the included study participants was obtained from WebRehab´s database. The Functional Independence Measure – FIM – measures level of disability and independence in two scales, one motor scale and one cognitive scale[25] and was analysed for description of ADL-functions and dependency.

To make certain that the data in WebRehab had been entered correctly (a validation), the medical charts were checked. To obtain charts, care units were contacted, first by letter, then

For peer review only - http://bmjopen.bmj.com/site/about/guidelines.xhtml

reminders were sent by email, and finally attempts were made to reach persons in charge by phone.

Data from the Swedish Tax Agency's population register were collected to investigate how many of the study persons were still alive and to obtain their addresses and contact information.

Data on cause of death and date of death were obtained from The National Board of Health and Welfare's registry on Cause of Death. Both the application and the communication following the application were written and handled by the first author.

During part two questionnaires and interviews were performed. An information letter along with the questionnaires was sent to all study persons still alive. If no response, a reminder was sent by letter and thereafter the attempts to reach the study persons or proxy was by phone. If the study persons wanted to participate, they were asked to send in the questionnaires or contact the author.

Four study persons who agreed to participate were visited for a personal, structured interview. The interviews were conducted in the participants' homes and were recorded and transcribed. The first author performed all interviews. During the interviews, a proxy gave some answers or a personal assistant and the rest were given by the participant and translated by next of kin. After the interview, the participant confirmed that the information given was correct, both the information given in the interview and in the questionnaires. One study participant was not able to perform a personal interview but participated through a telephone interview with her representative.

Questionnaires

For peer review only

The different questionnaires used were Stroke Impact Scale (SIS-v3.0)[26], RAND-36, Impact on Participation and Autonomy (IPA-E)[27] and EQ-5D briefly described below.

SIS-v3.0

To assess health status a Swedish version of the SIS-v3.0[28] was used. The SIS is developed for, validated, and shown to be reliable for stroke patients.[26 29]

RAND-36

RAND-36 is a survey instrument that assesses health-related Quality of Life. The Swedish version of RAND-36 is a modern translation of The Short form Health Survey – SF-36, but it is similar enough to allow comparisons. It is reliable and valid for measures on HRQoL in stroke patients.[30]

IPA-E

IPA-E was used to assess and measure impact on participation and autonomy. The original version of IPA has good validity and reliability[31] IPA-E includes 5 domains: autonomy indoors, family role, autonomy outdoors, social life and relationships, and work and education.[27]

EQ-5D

To assess Health Related Quality of Life (HRQoL), a Swedish version of the questionnaire EQ-5D was used. EQ-5D is a standardized, validated health questionnaire developed by the EuroQol Group Association.[32] It is intended to be used for self-completion in postal surveys, interviews and clinical practice.[33] The EQ-5D is a valid and reliable measure of HRQoL after stroke.[33] EQ-5D assesses health in 5 dimensions: mobility, self-care, usual activities, pain/discomfort, and anxiety/depression.

Statistical methods

For peer review only - http://bmjopen.bmj.com/site/about/guidelines.xhtml

For statistical analyses, IBM® *SPSS Statistics 21* was used. Mainly descriptive statistics with mean and median values were used for the demographic data.

.

For peer review only

Results

Population characteristics

The characteristics of the study population are summarized in table 1.

Table 1 Characteristics of the study population

Study persons	n=10
Alive	*7*
Deceased	*3*
Cause of LiS	
Ischemic stroke	*7*
Haemorrhagic stroke	*3*
Classification of LiS at admittance for rehabilitation	
Classic	*6*
Incomplete	*4*
Previous CVD/VRF	6
Total in hospital stay Median (Range)	216 days (150-627)
Length of Stay in Rehab Median (Range)	151 days (63-345)
Age at onset Median (Range)	49 y (22-67)
Survival* Median (Range)	1.9 y (1.5-2.3)
Time since onset** Median (Range)	5.9 y (2.3-8.1)

** Deceased study persons only included*

*** Alive study persons only included*

LiS - Locked-in syndrome, CVD - Cardiovascular disease, VRF - Vascular Risk Factors

Out of ten study persons, there were three women and seven men; age at onset varied from 22 years to 67 years with a median of 49 years. In six of the cases of ischemic stroke, the underlying cause was a basilar thrombosis, and the seventh case a vertebral artery dissection. Six persons had cardiovascular disease or documented vascular risk factors. The most

For peer review only

common vascular risk factor was hypertension. The patients were admitted to intensive care and then at stroke units before the rehabilitation period. The length of stay in hospital? before rehab varied between the hospitals and was not always dependent on the medical needs of the patient. Nine of the study persons experienced respiratory complications during hospitalization.

No change in ADL-dependency in motor scale domains was seen in any of the study persons when measured with FIM at admission to rehab and discharge; all were completely dependent in all domains. However, the social-cognitive part of FIM improved with rehabilitation since communication was improved (which has an impact in these items).

Seven persons from the total population (n=10) were still alive at start of study and three persons were deceased. One person died during rehabilitation and the remaining two after the initial rehabilitation period, time from onset to date of death was 1.5, 1.9 and 2.3 years. The cause of death was pulmonary embolism, acute myocardial infarction, and acute vascular disorders of the intestine. In none of the cases was the cause of death reported as being the result of respiratory complication due to LiS.

For details of all study persons and of participants, see tables 2 and 3

For peer review only

Table 2. All study persons

	Study persons									
	2	3	4	5	6	7	8	9	10	11
Aetiology	Vascular [illegible]	Vascular [illegible]	Vascular (I)	Vascular [illegible]	Vascular (I)	Vascular (H)	Vascular (I)	Vascular (H)	Vascular (I)	Vascular [illegible]
Classification	Classic	Classic	Incomplete	Classic	Classic	Incomplete	Incomplete	Incomplete	Classic	Classic
Discharged to	Nursing home	Short-term care	Short-term care	Nursing home	Independent living with personal assistance	Independent living with personal assistance	Died during rehab	Independent living with personal assistance	Short-term care	Nursing home
Survival	1	1.9 y	1	2.3 y	1	1	1.6 y	1	1	1
Cause of death (ICD-10-SE)		Pulmonary embolism (I26.9)		Acute vascular disorders of the intestine (K55.0)			Acute myocardial infarction (I21.9)			
Present form of residency	Nursing home		Apartment with community-based support		Independent living with personal assistance	Independent living with personal assistance		Independent living with personal assistance	Independent living with personal assistance	Nursing home

1 Still alive

Abbreviations: H - Haemorrhagic, I – Ischemic

Table 3. Assistance to and communication type of participants

	Followed up patients			
	2	4	6	10
Time since LiS	8 years	7.5 years	5.5 years	7 years
Living arrangement	Lives in a nursing home/home with society-based support. Joint common areas.	Lives in an apartment with society-based support. Lives alone.	Lives in a house. Lives with family.	Lives in a house. Lives with family.
Personal assistance	No personal assistants. Gets assistance from staff.	External personal assistants + extra assistance from staff when needed night-time	External personal assistance + partner as paid assistant	External personal assistance + partner as paid assistant
Amount	Assistance from staff 168h/week	Assistance - 168 h/week<br>Dual staffing - 10.5 h/week<br>Total – 178.5 h/week	Single staffing - 168 h/week.	Assistance - 168 h/week<br>Dual staffing - 80.5 h/week<br>Total – 248.5 h/week
Communication	Alphabet board, blinking	Mainly alphabet board (blinking sometimes)	Oral, alphabet board (pointing)	Blinking, eye-tracking device
Nutrition	Percutaneous endoscopic gastrostomy (PEG)	Oral, pureed/semi-solid	Oral, solid diet	PEG, occasional treat by mouth
Locomotion	Electric wheelchair	Electric wheelchair	Electric/manual wheelchair. Can walk a few steps with support	Electric wheelchair

For peer review only

HRQoL and impact on participation

Results from the questionnaires are presented separately for each questionnaire. No mean scores were calculated due to the low number of participants in the follow-up. To put the participants' scores in perspective, values for reference populations are included in the tables. The main finding from the questionnaires is that, although values vary between the follow-up participants, higher scores were seen in cognitive and mental domains (e.g. SIS-v3.0 Memory and Emotion and RAND-36 Mental Health) than in physical domains (e.g. SIS-v3.0 Strength, RAND-36 Physical Functioning and EQ-5D Mobility).

Individual scores on the SIS-v3.0 are presented in table 4, on RAND-36 in table 5, on IPA-E in table 6 and on EQ-5D in table 7.

Table 4 Stroke Impact Scale Results

	**Individual score**				**Reference*** Mean *(SD)*
**Domain**	**2**	**4**	**6**	**10**	
Strength	25	37.5	25	0	71 *(26.9)*
Hand function	0	0	30	0	81.1 *(22.2)*
Mobility	0	0	19.4	5.6	77.6 *(17.4)*
ADL	0	12.5	15	20	85 *(21)*
Emotion	44.4	94.4	88.9	86.1	75 *(28.5)*
Memory	100	42.9	100	71.4	75.6 *(29.2)*
Communication	75	85.7	53.6	14.3	68.9 *(34.5)*
Social participation	12.5	31.25	78.1	25	70.3 *(27.5)*
Stroke recovery	20	50	100	10	68.4 *(25.5)*

* Swedish stroke population, assessed 12 months after stroke.[28]

For peer review only - http://bmjopen.bmj.com/site/about/guidelines.xhtml

Table 5 RAND-36 Results

Domain	Individual score 2	4	6	10	Reference (LiS)* Mean *(SD)*	Reference (Stroke)** Mean *(95% CI)*
Physical functioning	0	0	5	0	0 *(0)*	51.2 *(44.3-58.1)*
Role limitations due to physical health	0	0	100	0	59.4 *(32.6)*	14.7 *(3.3-26.1)*
Role limitations due to emotional problems	33.3	100	100	100	75.0 *(34.5)*	18.0 *(10.2-25.8)*
Vitality/Energy-fatigue	45	55	75	70	64.4 *(24.6)*	42.9 *(37.8-48.0)*
Mental health/Emotional well-being	60	76	92	76	68 *(19.6)*	62.7 *(58.2-67.2)*
Social functioning	12.5	75	50	87.5	56.3 *(34.1)*	55.2 *(49.2-61.4)*
Bodily pain	80	90	100	67.5	82 *(26.8)*	65.0 *(57.9-72.1)*
General health	40	30	100	60	63.5 *(33.0)*	58.2 *(52.5-63.9)*

* Belgian LiS population, assessed more than 12 months after onset.[11]
** Swedish stroke population, assessed 2 years after day hospital rehabilitation for stroke.[34]

Table 6 Impact on Participation and Autonomy Questionnaire Results

Domain	Individual score 2	4	6	10	Reference * Mean *(SD)*
Autonomy indoors	4	3	0	2	0.96 *(0.6)*
Family role	4	4	4	4	1.96 *(1.1)*
Autonomy outdoors	4	3	2	3	2.35 *(0.9)*
Social life and relations	3	2	3	3	1.48 *(0.7)*
Work and education	-[1]	-[1]	1	-[1]	-

[1]Cannot be assessed, participant is not currently employed.
*Iranian stroke population, assessed 5-36 months after their stroke.[35]

For peer review only - http://bmjopen.bmj.com/site/about/guidelines.xhtml

Table 7 EuroQol-5 dimensions Results

	Individual score				Reference*
**Domain**	**2**	**4**	**6**	**10**	Mean *(95% CI)*
Mobility	3	3	3	3	-
Self-care	3	3	3	3	-
Usual activities	3	3	1	3	-
Pain/discomfort	3	2	1	2	-
Anxiety/depression	2	2	1	2	-
Index value	-0.429	-0.166	0.122	-0.166	0.44 *(0.38-0.50)*
VAS	0.02	0.5	1	0.4	62.7 *(58.8-66.6)*

*Swedish stroke population, assessed 2 years after day hospital rehabilitation for stroke.[34]

Interviews

Information

In all but one interview, the follow-up participants described a lack of information about LiS and it was difficult to get information about technical devices, or the follow-up procedure. They mentioned that they were sometimes forced to find technical aids and organize follow-up on their own. One participant, and a proxy, intended to write a book for persons with LiS about how to get information and support. This participant also found this information and support by becoming a member of a patient association.

When the follow-up participants were in intensive care, they felt they got better information than after discharge, although medical terms were sometimes difficult to understand.

Technical devices, technical adjustments and shortcomings

All used one or more wheel-chair/s; alphabet boards and used or wanted to use an eye-tracking computer device. This kind of computer is expensive and was not available for everyone.

For peer review only - http://bmjopen.bmj.com/site/about/guidelines.xhtml

For peer review only

An alarm button which can be placed by the temple, adjusted hospital beds and lifts, tilting table, ceiling hoist, a walking frame with extra support and a sit-to stand and transfer device were also mentioned as technical devices that the follow-up participants had access to. Two used bicycles for passive cycling. One person wanted a Functional Electric Stimulation/FES-assisted training device, but the municipality refused funding. This person was also part of a customer test group for a device which combines eye-tracking technology with an electric wheelchair to enable control of the wheelchair with eye movements. He was promised money to purchase a wheelchair because of his help in developing the new technology.

Adjustments of homes were made, such as installing ramps, widening doors, thresholds and elevated toilet seats.

Progress after rehabilitation and care

Several years after stroke, the follow-up participants' motor functions were still improving. They could communicate with the help of an alphabet board and used an eye-tracking computer device, or communicated mainly by blinking. One person had some oral communication including most vowels, some consonants and a few short words at discharge. At the time of assessment, he could control his jaw and tongue muscles and he had some movement in his fingers. Another could walk a few steps with support, lift his arms and move his head; he also had some function in his left hand, but was still completely dependent in ADL. Two persons could eat solid food and had no additional nutritional support. One person had an alarm button with a pre-recorded sentence, which he could press by turning his head to attract attention. During the last year, he had learnt to shake his head and is currently practicing nodding. All needed a lot of daily support and had personal assistants around the clock and sometimes two assistants at the same time.

For peer review only - http://bmjopen.bmj.com/site/about/guidelines.xhtml

Likes and dislikes about care and authorities

The interviewed persons with LIS appreciated having phenomenal physiotherapists during the rehabilitation period. Every second week both an occupational therapist and a physiotherapist visited one participant, she still wanted a lot more additional training on top of these visits. Some said contacts with authorities mostly worked well, although it could be time-consuming, but one argued that daily contacts with caregivers worked less well. One participant felt unhappy because she was in a nursing home and could not live on her own terms but was granted a flat of her own in due time.

Some follow-up participants/ proxies were offered support by a counsellor and were pleased with this. One partner talked about the difficult mourning process after LiS. Nothing turned out the way she had wanted because of her husband´s LiS. She was helped to think and reflect in a new way, such as what could be planned and done despite LiS. One partner reflected that during intense rehabilitation periods, one gets a lot of support from all specialists but then it ends, for example appointments with a counsellor. One person wanted to meet a specialist doctor but felt he was not heard, having so many questions not answered by the local doctor.

Attitudes from professionals

All interviewed experienced not being treated as adults, people talked to them slowly and loudly as if they were not able to understand anything which was frustrating. This could be aggravating if a person treating you did not know you well enough and/or did not have enough knowledge about the syndrome. A proxy said that just being able to nod your head led to a participant being treated with more respect.

For peer review only

For peer review only - http://bmjopen.bmj.com/site/about/guidelines.xhtml

Family and friends

One participant had separated from her husband and had a complicated life situation. The others had mostly good family relationships, in two cases the wives worked as personal assistants. It was articulated that a relationship with a daughter had worsened after LiS and that this was the worst thing that had happened. Friends might have disappeared after LiS but the remaining ones were appreciated.

Quality of life

All but one evaluated their current life situation. They mentioned feeling joy at being alive and having the ability to encourage others. Improving quality of life could mean being granted more hours with personal assistants or getting in contact with a person with the same syndrome.

Discussion

Findings

We identified ten persons who had been diagnosed with LiS between 2007 and 2014 and investigated factors at onset, during the rehabilitation period, and after discharge. Seven of these persons were still alive at the start of the study and four of these participated. The common cause was stroke and most of these were ischemic. According to medical charts, some of the study persons improved in their motor function and all are still improving, although not in independency measured with the FIM motor scale. No major cognitive deficits were reported. Mortality in this population was 30 % and mean survival time for the deceased was 1.9 years. For the study persons still alive, mean time since onset of LiS was 5.9 years. From the interviews, we identified areas with unfulfilled needs: information, respect,

For peer review only - http://bmjopen.bmj.com/site/about/guidelines.xhtml

For peer review only

and specialized technical aids. The data from the questionnaires were mostly in line with information from the interviews: those who expressed high QoL and high impact on participation and autonomy in the interview, also scored high in domains not affected by motor impairments in the questionnaires.

Strengths and limitations

Since this was a nation-wide study and no selection was done, the number of patients might be considered few compared to previous studies abroad with study samples of around 20-30 persons.[5 13 36] One hypothesis could be that incidence numbers on LiS differ for some reason in Sweden. Another could be that incidence numbers are similar but not all persons with LiS in Sweden have been identified. This study population was identified through a register for patients having been admitted to rehabilitation medicine units and therefore, did not include patients who died in intensive care units. Up until the end of 2014, WebRehab was, to our knowledge, the only register in Sweden that had statistics on LiS but as of 1st of January 2015, there is an ICD-code in ICD-10-SE. What this will mean for the care of LiS-patients is hard to predict but reporting LiS will be possible for every care unit.

The questionnaires used are similar to each other in many ways, but all of them include unique aspects when compared to each other. EQ-5D is a quite rough instrument and physical functions have a large impact on the result. The value of using it in this particular study, where physical functions are very low, might therefore be worth discussing. In the EQ-5D, an index score under 0 is described as indicating a condition worse than death which is a problematic statement. For instance, three of our follow-up participants had an index score under 0, but none of them described their situation as worse than death.

When presenting the results of the questionnaires, values from reference populations are included in the tables, and our reasoning was to put our participants' scores in perspective. However, due to the low numbers of follow-up participants, no further comparisons can be

For peer review only - http://bmjopen.bmj.com/site/about/guidelines.xhtml

made. The reference groups are all stroke populations, because all our participants had suffered a stroke and are included in this patient group.

The method chosen for this study was a quantitative, descriptive method containing analysis of register data, medical charts and structured interviews based on questionnaires. The reason we chose this approach instead of a qualitative method with unstructured interviews was the follow-up participants' limited ability to communicate effectively. This we believe would mean unstructured interviews would not give more information than structured interviews. By combining questionnaires with personal interviews, the follow-up participants had a chance to elaborate on their answers and opinions while still maintaining a standardized form of assessment with the questionnaires.

Since a proxy or a personal assistant was present and involved in the interviews, there is a risk of misinterpretations in translations or that things were not relayed in the way the participant intended. All follow-up participants were therefore asked if the information given was correct at the end of the interviews.

Comparison with existing literature and guidelines

It seems consistent with previous knowledge that mortality is high during the first years after onset but when stabilized, persons with LiS may live for decades.[7] Since onset of LiS for our study population only goes back to 2007, real long-term survival cannot be commented upon here.

The cause of death of the study persons was never a result of respiratory complications or problems with breathing, despite the high prevalence of respiratory complications during hospitalization. This might be explained by proper care, which has prevented or successfully treated any respiratory problems, but it might also be explained by chance.

The importance of maintaining vital functions and that fatality in LiS has declined with improvements in quality of medicine has been discussed in previous studies.[6] In line with

For peer review only - http://bmjopen.bmj.com/site/about/guidelines.xhtml

previous findings,[7 10 11] most of the study persons were middle-aged with a median age of 49 years at onset. In line with our study, patients with chronic LiS have slim chances of major improvements in motor function.[10]

According to FIM, five persons improved in independency in cognitive domains. Expression and problem solving are the two areas with the least improvements, which might be explained by the poor communication skills of this patient group.

In many aspects, the results on the questionnaires varied between the participants but most of them scored high on domains measuring emotional and cognitive functions and low on physical domains and functions. This is in line with previous knowledge that QoL in persons with LiS can be high scoring in mental domains, while low scoring in physical domains.[13]

Clinical and policy implications

Problems like nursing staff using complicated medical terminology or not getting enough information about technical and rehabilitation aids were brought up. As this patient group has limited communication skills, it seems important to fulfil all their needs for technical equipment to improve their QoL. All participants had experienced that they were not being treated with respect, and felt that people talked over their heads. These problems have been discussed in context of disability and aphasia, it affects social participation and with that, QoL in a negative way.[37 38] Raising awareness about LiS, both among caregivers and the public is necessary, since it might lead to less insecurity and incertitude when meeting a person with LiS and, therefore result in people treating a person with LiS with more respect.

Future research

From 2015, there will be an ICD-code in ICD-10-SE, which will improve the possibility of longitudinal research of this group of patients in Sweden. This seems to be an urgent field of

For peer review only - http://bmjopen.bmj.com/site/about/guidelines.xhtml

research because, to our knowledge, there are no articles yet from Sweden about LiS. In addition, improving the development and research of new technical devices for this group of patients might help them to communicate more efficiently and to find methods of improving motor functions.

Author contribution

KS, ÅN and KSS contributed to the design of the study. KS met the participants and gave them questionnaires, conducted and summarized the structured interviews. MT categorized the interviews inductively and wrote the manuscript together with KS and KSS.

Data sharing statement

Due to ethical restrictions, data are available upon request. Interested researchers may submit requests for data to the authors (contact Professor Katharina S. Sunnerhagen, email: ks.sunnerhagen@neuro.gu.se). According to Swedish regulation: http://www.epn.se/en/start/regulations/, the permission to use data is only for what has been applied for and then approved by the Ethical board. Not following the regulations is seen as scientific misconduct.

Competing interests

All authors declare that there is no conflict of interest.

Acknowledgements

We would like to acknowledge the help from WebRehab and the clinics that provided information on the persons. At finally yet importantly, we would like to thank the participants for answering questionnaires. Sincere thanks to them and their families for allowing KS to visit them and for generously telling about their situation and experiences. We would like to thank Dr Kate Bramley-Moore for her English language assistance and proofreading.

For peer review only - http://bmjopen.bmj.com/site/about/guidelines.xhtml

Funding

The study is in part funded by The Swedish Research Council (2017-00946) and a grant from the Swedish Heart and Lung Foundation (20160526). The supporting bodies had no influence on the content of the study.

Patient and Public Involvement

In the steering committee for the national quality register WebRehab, there are two representatives from patient organizations. There were no patient representatives involved in the recruitment to or conduction of the study. A short summary of the results has been distributed to the follow-up participants in the interviews.

For peer review only

References

1. Posner JB, Saper CB, Schiff ND, et al. Plum and Posner's Diagnosis of Stupor and Coma. 4 ed. New York: Oxford University Press 2007.
2. Patterson JR, Grabois M. Locked-in syndrome: a review of 139 cases. *Stroke* 1986;17(4):758-64. [published Online First: 1986/07/01]
3. Schjolberg A, Sunnerhagen KS. Unlocking the locked in; a need for team approach in rehabilitation of survivors with locked-in syndrome. *Acta Neurol Scand* 2012;125(3):192-8. doi: 10.1111/j.1600-0404.2011.01552.x [doi] [published Online First: 2011/06/29]
4. Doble JE, Haig AJ, Anderson C, et al. Impairment, activity, participation, life satisfaction, and survival in persons with locked-in syndrome for over a decade: follow-up on a previously reported cohort. *J Head Trauma Rehabil* 2003;18(5):435-44. [published Online First: 2003/09/16]
5. Katz RT, Haig AJ, Clark BB, et al. Long-term survival, prognosis, and life-care planning for 29 patients with chronic locked-in syndrome. *Arch Phys Med Rehabil* 1992;73(5):403-8. doi: 0003-9993(92)90025-R [pii] [published Online First: 1992/05/01]
6. Nikic PM, Jovanovic D, Paspalj D, et al. Clinical characteristics and outcome in the acute phase of ischemic locked-in syndrome: case series of twenty patients with ischemic LIS. *Eur Neurol* 2013;69(4):207-12. doi: 000345272 [pii]
10.1159/000345272 [doi] [published Online First: 2013/01/12]
7. Leon-Carrion J, van Eeckhout P, Dominguez-Morales Mdel R. The locked-in syndrome: a syndrome looking for a therapy. *Brain Inj* 2002;16(7):555-69. doi: 10.1080/02699050110119466 [doi] [published Online First: 2002/07/18]
8. American Congress of Rehabilitation M. Recommendations for use of uniform nomenclature pertinent to patients with severe alterations in consciousness. *Archives of Physical Medicine and Rehabilitation* 1995;76(2):205-09.
9. Pearce JM. The locked in syndrome. *Br Med J (Clin Res Ed)* 1987;294(6566):198-9. [published Online First: 1987/01/24]
10. Casanova E, Lazzari RE, Lotta S, et al. Locked-in syndrome: improvement in the prognosis after an early intensive multidisciplinary rehabilitation. *Arch Phys Med Rehabil* 2003;84(6):862-7. doi: S000399930300008X [pii] [published Online First: 2003/06/17]
11. Snoeys L, Vanhoof G, Manders E. Living with locked-in syndrome: an explorative study on health care situation, communication and quality of life. *Disabil Rehabil* 2013;35(9):713-8. doi: 10.3109/09638288.2012.705950 [doi] [published Online First: 2012/08/18]
12. Soderholm S, Meinander M, Alaranta H. Augmentative and alternative communication methods in locked-in syndrome. *J Rehabil Med* 2001;33(5):235-9. [published Online First: 2001/10/05]
13. Rousseau MC, Pietra S, Nadji M, et al. Evaluation of quality of life in complete locked-in syndrome patients. *Journal of Palliative Medicine* 2013;16(11):1455-58.
14. Parvizi J, Anderson SW, Martin CO, et al. Pathological laughter and crying: A link to the cerebellum. *Brain* 2001;124(9):1708-19.
15. Barbic D, Levine Z, Tampieri D, et al. Locked-in syndrome: a critical and time-dependent diagnosis. *CJEM* 2012;14(5):317-20. [published Online First: 2012/09/13]
16. Janjua N, Wartenberg KE, Meyers PM, et al. Reversal of locked-in syndrome with anticoagulation, induced hypertension, and intravenous t-PA. *Neurocrit Care* 2005;2(3):296-9. doi: NCC:2:3:296 [pii]

For peer review only - http://bmjopen.bmj.com/site/about/guidelines.xhtml

10.1385/NCC:2:3:296 [doi] [published Online First: 2005/09/15]
17. World Health Organization. International Classification of Functioning, Disability and Health (ICF). Geneva, 2001.
18. Cardol M, De Jong BA, Ward CD. On autonomy and participation in rehabilitation. *Disability and Rehabilitation* 2002;24(18):970-74.
19. Albrecht GL, Devlieger PJ. The disability paradox: High quality of life against all odds. *Social Science and Medicine* 1999;48(8):977-88.
20. Ubel PA, Schwarz N, Loewenstein G, et al. Misimagining the unimaginable: The disability paradox and health care decision making. *Health Psychology* 2005;24(4 SUPPL.):S57-S62.
21. Asplund K, Hulter Asberg K, Appelros P, et al. The Riks-Stroke story: building a sustainable national register for quality assessment of stroke care. *Int J Stroke* 2011;6(2):99-108. doi: 10.1111/j.1747-4949.2010.00557.x [doi] [published Online First: 2011/03/05]
22. Sunnerhagen KS, Flansbjer UB, Lannsjo M, et al. WebRehab: a Swedish database for quality control in rehabilitation. *J Rehabil Med* 2014;46(10):958-62. doi: 10.2340/16501977-1886 [doi] [published Online First: 2014/10/10]
23. Association du Locked in Syndrome ALIS. LISEF LIS European Foundation [Internet]. [cited 2015 26th May]. Available from: http://www.alis-asso.fr/lisef/ accessed May 26th 2015.
24. Giacino JT, Ashwal S, Childs N, et al. The minimally conscious state: Definition and diagnostic criteria. *Neurology* 2002;58(3):349-53.
25. Grimby G, Gudjonsson G, Rodhe M, et al. The functional independence measure in Sweden: experience for outcome measurement in rehabilitation medicine. *Scand J Rehabil Med* 1996;28(2):51-62. [published Online First: 1996/05/01]
26. Duncan PW, Wallace D, Lai SM, et al. The stroke impact scale version 2.0. Evaluation of reliability, validity, and sensitivity to change. *Stroke* 1999;30(10):2131-40. [published Online First: 1999/10/08]
27. Kersten P, Cardol M, George S, et al. Validity of the impact on participation and autonomy questionnaire: a comparison between two countries. *Disabil Rehabil* 2007;29(19):1502-9. doi: 771341328 [pii]
10.1080/09638280601030066 [doi] [published Online First: 2007/09/14]
28. Guidetti S, Ytterberg C, Ekstam L, et al. Changes in the impact of stroke between 3 and 12 months post-stroke, assessed with the Stroke Impact Scale. *J Rehabil Med* 2014;46(10):963-8. doi: 10.2340/16501977-1865 [doi] [published Online First: 2014/09/05]
29. Duncan PW, Bode RK, Min Lai S, et al. Rasch analysis of a new stroke-specific outcome scale: the Stroke Impact Scale. *Arch Phys Med Rehabil* 2003;84(7):950-63. doi: S0003999303000352 [pii] [published Online First: 2003/07/26]
30. Anderson C, Laubscher S, Burns R. Validation of the Short Form 36 (SF-36) health survey questionnaire among stroke patients. *Stroke* 1996;27(10):1812-16.
31. Larsson Lund M, Nordlund A, Nygard L, et al. Perceptions of participation and predictors of perceived problems with participation in persons with spinal cord injury. *J Rehabil Med* 2005;37(1):3-8. doi: W4GD12KB4ELWXC0T [pii]
10.1080/16501970410031246 [doi] [published Online First: 2005/03/25]
32. EuroQol Research Foundation. [Internet]. Rotterdam: EuroQol Research Foundation; 2015 [Available from: http://www.euroqol.org/about-eq-5d.html accessed Feb 6 2015.
33. Dorman PJ, Waddell F, Slattery J, et al. Is the EuroQol a valid measure of health-related quality of life after stroke? *Stroke* 1997;28(10):1876-82. [published Online First: 1997/10/28]

For peer review only - http://bmjopen.bmj.com/site/about/guidelines.xhtml

34. Grenthe Olsson B, Sunnerhagen KS. Functional and cognitive capacity and health-related quality of life 2 years after day hospital rehabilitation for stroke: a prospective study. *J Stroke Cerebrovasc Dis* 2007;16(5):208-15. doi: S1052-3057(07)00079-1 [pii]
10.1016/j.jstrokecerebrovasdis.2007.06.002 [doi] [published Online First: 2007/09/12]
35. Fallahpour M, Tham K, Joghataei MT, et al. Perceived participation and autonomy: aspects of functioning and contextual factors predicting participation after stroke. *J Rehabil Med* 2011;43(5):388-97. doi: 10.2340/16501977-0789 [doi] [published Online First: 2011/03/31]
36. Leon-Carrion J, van Eeckhout P, Dominguez-Morales Mdel R, et al. The locked-in syndrome: a syndrome looking for a therapy. *Brain Inj* 2002;16(7):571-82. doi: 10.1080/02699050110119781 [doi] [published Online First: 2002/07/18]
37. Dalemans RJ, de Witte L, Wade D, et al. Social participation through the eyes of people with aphasia. *Int J Lang Commun Disord* 2010;45(5):537-50. doi: 10.3109/13682820903223633 [pii]
10.3109/13682820903223633 [doi] [published Online First: 2009/10/21]
38. Wagner M. "Does he take sugar?". *World Health Forum* 1991;12(1):87-9. [published Online First: 1991/01/01]

For peer review only

For peer review only - http://bmjopen.bmj.com/site/about/guidelines.xhtml

Figure 1 legend

Flow chart of the study. One person was excluded after the validation process due to not meeting the inclusion criteria (locked in syndrome) and one person due to not having a valid personal identity number.

For peer review only

For peer review only - http://bmjopen.bmj.com/site/about/guidelines.xhtml

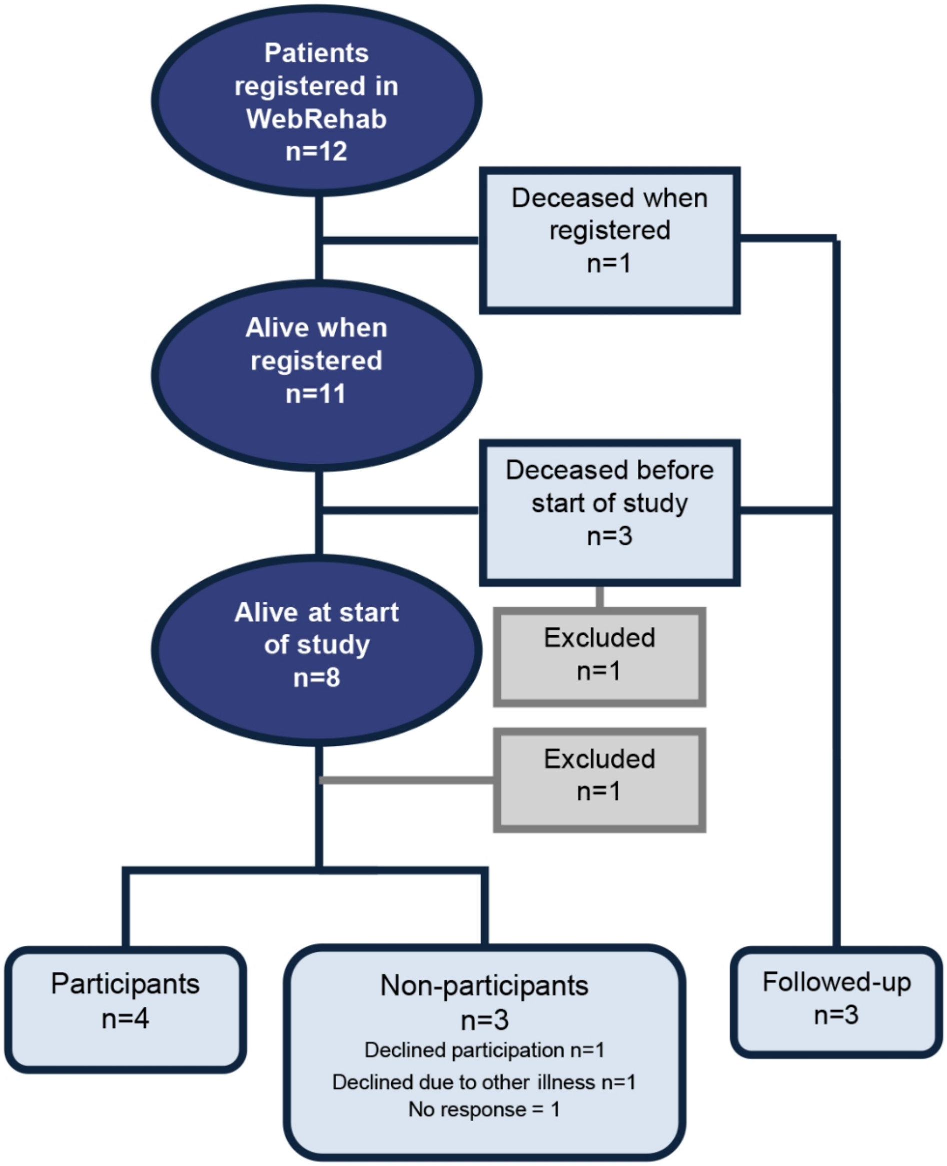

99x119mm (300 x 300 DPI)

STROBE 2007 (v4) Statement—Checklist of items that should be included in reports of *cohort studies*

**Section/Topic**	**Item #**	**Recommendation**	**Reported on page #**
**Title and abstract**	1	(*a*) Indicate the study's design with a commonly used term in the title or the abstract	1
		(*b*) Provide in the abstract an informative and balanced summary of what was done and what was found	2
**Introduction**			
Background/rationale	2	Explain the scientific background and rationale for the investigation being reported	4,5
Objectives	3	State specific objectives, including any prespecified hypotheses	6
**Methods**			
Study design	4	Present key elements of study design early in the paper	6
Setting	5	Describe the setting, locations, and relevant dates, including periods of recruitment, exposure, follow-up, and data collection	6,7
Participants	6	(*a*) Give the eligibility criteria, and the sources and methods of selection of participants. Describe methods of follow-up	7
		(*b*) For matched studies, give matching criteria and number of exposed and unexposed	NA
Variables	7	Clearly define all outcomes, exposures, predictors, potential confounders, and effect modifiers. Give diagnostic criteria, if applicable6	8.9
Data sources/ measurement	8*	For each variable of interest, give sources of data and details of methods of assessment (measurement). Describe comparability of assessment methods if there is more than one group	*8,9*
Bias	9	Describe any efforts to address potential sources of bias	NA
Study size	10	Explain how the study size was arrived at	8,9
Quantitative variables	11	Explain how quantitative variables were handled in the analyses. If applicable, describe which groupings were chosen and why	9
Statistical methods	12	(*a*) Describe all statistical methods, including those used to control for confounding	9
		(*b*) Describe any methods used to examine subgroups and interactions	NA
		(*c*) Explain how missing data were addressed	NA
		(*d*) If applicable, explain how loss to follow-up was addressed	NA
		(*e*) Describe any sensitivity analyses	NA
**Results**			

For peer review only

Participants	13*	(a) Report numbers of individuals at each stage of study—eg numbers potentially eligible, examined for eligibility, confirmed eligible, included in the study, completing follow-up, and analysed	9
		(b) Give reasons for non-participation at each stage	9
		(c) Consider use of a flow diagram	Fig 1
Descriptive data	14*	(a) Give characteristics of study participants (eg demographic, clinical, social) and information on exposures and potential confounders	10,12
		(b) Indicate number of participants with missing data for each variable of interest	10,12
		(c) Summarise follow-up time (eg, average and total amount)	
Outcome data	15*	Report numbers of outcome events or summary measures over time	12, 14.15,16
Main results	16	(*a*) Give unadjusted estimates and, if applicable, confounder-adjusted estimates and their precision (eg, 95% confidence interval). Make clear which confounders were adjusted for and why they were included	
		(*b*) Report category boundaries when continuous variables were categorized	
		(*c*) If relevant, consider translating estimates of relative risk into absolute risk for a meaningful time period	
Other analyses	17	Report other analyses done—eg analyses of subgroups and interactions, and sensitivity analyses	16,17
**Discussion**			
Key results	18	Summarise key results with reference to study objectives	20
**Limitations**			
Interpretation	20	Give a cautious overall interpretation of results considering objectives, limitations, multiplicity of analyses, results from similar studies, and other relevant evidence	20
Generalisability	21	Discuss the generalisability (external validity) of the study results	20
**Other information**			
Funding	22	Give the source of funding and the role of the funders for the present study and, if applicable, for the original study on which the present article is based	24

*Give information separately for cases and controls in case-control studies and, if applicable, for exposed and unexposed groups in cohort and cross-sectional studies.

Note: An Explanation and Elaboration article discusses each checklist item and gives methodological background and published examples of transparent reporting. The STROBE checklist is best used in conjunction with this article (freely available on the Web sites of PLoS Medicine at http://www.plosmedicine.org/, Annals of Internal Medicine at http://www.annals.org/, and Epidemiology at http://www.epidem.com/). Information on the STROBE Initiative is available at www.strobe-statement.org.

For peer review only
